# Moderate mechanical stimulation rescues degenerative annulus fibrosus by suppressing caveolin-1 mediated pro-inflammatory signaling pathway

**DOI:** 10.7150/ijbs.57774

**Published:** 2021-04-03

**Authors:** Weidong Zhang, Huan Wang, Zhangqin Yuan, Genglei Chu, Heng Sun, Zilin Yu, Huan Liang, Tao Liu, Feng Zhou, Bin Li

**Affiliations:** 1 Department of Orthopaedic Surgery, Orthopaedic Institute, The First Affiliated Hospital, Soochow University, Suzhou, Jiangsu, China.; 2 China Orthopaedic Regenerative Medicine Group (CORMed), Hangzhou, Zhejiang, China.

**Keywords:** annulus fibrosus, moderate mechanical stimulation, annulus fibrosus regeneration, caveolin-1, integrin β1, NF-κB, anti-inflammation

## Abstract

Mechanical loading can induce or antagonize the extracellular matrix (ECM) synthesis, proliferation, migration, and inflammatory responses of annulus fibrosus cells (AFCs), depending on the loading mode and level. Caveolin-1 (Cav1), the core protein of caveolae, plays an important role in cellular mechanotransduction and inflammatory responses. In the present study, we presented that AFCs demonstrated different behaviors when subjected to cyclic tensile strain (CTS) for 24 h at a magnitude of 0%, 2%, 5% and 12%, respectively. It was found that 5% CTS had positive effects on cell proliferation, migration and anabolism, while 12% CTS had the opposite effects. Besides, cells exposed to interleukin-1β stimulus exhibited an increase expression in inflammatory genes, and the expression of these genes decreased after exposure to moderate mechanical loading with 5% CTS. In addition, 5% CTS decreased the level of Cav1 and integrin β1 and exhibited anti-inflammatory effects. Moreover, the expression of integrin β1 and p-p65 increased in AFCs transfected with Cav1 plasmids. *In vivo* results revealed that moderate mechanical stimulation could recover the water content and morphology of the discs. In conclusion, moderate mechanical stimulation restrained Cav1-mediated signaling pathway and exhibited anti-inflammatory effects on AFCs. Together with *in vivo* results, this study expounds the underlying molecular mechanisms on the effect of moderate mechanical stimulation on intervertebral discs (IVDs) and may provide a new therapeutic strategy for the treatment of IVD degeneration.

## Introduction

Degenerative disc disease (DDD) is a global public health problem caused by several factors and represents a leading cause of disability 1. Years lived with disability (YLDs) caused by DDD increased by 54% from 1990 to 2015 and continue to grow due to the aging of the population 2. The social expenditure caused by the lost productivity, dispersal of disability benefits and medical cost might top $200 billion annually 3. Therefore, treatment of DDD has tremendous economic and social impact. Surgical therapies usually provide fast relief from serious back pain or leg numbness symptom in patients with DDD, but tend to accelerate the degeneration of adjacent discs 4. However, the clinical symptoms of the patients with early degeneration treated by conservative methods diminish or completely disappear in several weeks, and rarely recur shortly 5. Taken together, conservative approach is the first choice for the management of the disc degeneration processes, especially in the phase of the DDD recognized as early stage.

The etiology of DDD is multi-factorial, resulting from combinatory effects of the genetic predisposition, aging, excessive mechanical load, traumatic injury, etc. 6, 7. In general, excessive load is considered as the main cause of DDD. Excessive spinal loading caused by faulty lifestyle or obesity can lead to metabolic disorders of the extracellular matrix (ECM) of intervertebral discs (IVD) 8. In addition, excessive spinal loading elevates the expression of pro-inflammatory genes, such as *Cox2*, interleukin-6 (*Il6*) and interleukin-8 (*Il8*) 9. Inflammation is mostly seen as a detrimental factor and involved in the onset of DDD 10, 11. In degenerative IVDs, the infiltration of activated immunocytes, including macrophages, T-cells, B-cells and natural killer cells, occurs in response to the production of several chemokines secreted by IVD cells and results in loss of ECM structural integrity of the disc 12, 13. Therefore, it is critical to relieve the inflammatory responses to restore IVD structure and functions.

IVD is a heterogeneous multi-component structure, which is composed of three distinct tissues, i.e., nucleus pulposus (NP), annulus fibrosus (AF) and cartilage endplate (CEP) 14. As an important component of IVD, AF can buffer the mechanical loads resulted from the rotation or curvature of spine and help prevent NP herniation. Mechanical forces are important modulators of AF degeneration. Haglund et al. reported that excessive mechanical stimulation accelerated ECM degeneration and drove inflammatory responses of AF tissues 15. Rannou et al. found that excessive mechanical stimulation could decrease the production of proteoglycans by AF, which led to dehydration of disc 16. In addition, abnormal mechanical loads (e.g., torsion, bending, flexion, etc.) also result in disc degeneration by directly affecting IVD cell metabolism and causing structural damage 17, 18. However, mechanical stimulation at the physiological level has been proposed as a therapeutic option for tissue repair and regeneration. Likewise, mechanical signals have been reported to promote stem cell proliferation and differentiation, and increasing evidence has suggested that mechanical cues play a key role in regulating AF repair and regeneration 19. Haglund et al. found that mechanical stimulation with physiological level was essential for AF health, by providing anabolic and anti-inflammatory effects 20. Schnake et al. summarized that physiologically equivalent stimulation might accelerate matrix synthesis and restore proteoglycan content, which had reparative effects on mildly degenerate IVD 21. Mechanical lumbar traction has been commonly used to treat patients with early disc degeneration since the middle of last century 22. It is reported that moderate traction contribute to the regeneration or repair of the disc by re-organizing the lamellar architecture of the annulus 23. Despite various explorations in this area, understanding of the mechanisms underlying tissue repair and regeneration induced by moderate mechanical stimulation remains in the nascent stages.

Integrin is a kind of heterodimeric membrane protein that is composed of α- and β-subunits 24. It acts as a transmembrane signaling molecule, relaying cues from the extracellular microenvironment to regulate cellular behaviors 25. It has been reported that the activation of integrin β1 signaling resulted in catabolism and inflammation of IVD during degenerative progression induced by static compression 26. Sokabe et al. found that integrin signaling pathway regulates mechanotransduction through the nuclear translocation of nuclear factor κB (NF-κB), which indicates the activate state of NF-κB family 27. And increased activation of NF-κB accelerates the transcription of inflammatory and catabolic genes such as *Il6*, *Il8*, Cox2,* Adamts4* and *Adamts5* 28, 29. Moreover, activation of NF-κB acts a central role in tension-related degenerative changes in articular cartilage and endplate, and elevated NF-κB activity can be observed in degenerative discs 30-32. However, how mechanical stimulation controls integrin β1 expression levels and NF-κB activity in degenerative discs remains largely unclear. Recent studies have discovered that caveola, a flask-shaped and invaginate structure at the cell surface, can conduct and coordinate various signals at the plasm membrane 33, 34. Caveolae exist widely in the tissues of heart, liver, kidney, skeletal, blood vessels and IVD, and are highly expressed in fibroblasts, chondrocytes, adipocytes, vascular endothelial cells, epithelial cells, etc. 35. Caveolae are involved in various biological functions, including mechano-sensing, ECM remodeling, cell migration, cell signaling, lipid metabolism, and tissue repair 36. Caveolins, the major components of caveolae, mainly include three isoforms, i.e., caveolin-1 (Cav1), caveolin-2 (Cav2) and caveoin-3 (Cav3) 37-39. Cav1, the core protein of caveolae, functions as a mechano-sensor and mechano-transducer in response to various mechanical stimulations from the cellular microenvironment 40. For examples, Cav1 is involved in the integrin-mediated inflammatory signaling pathway by acting as a mechano-sensor which senses shear stress, pressure and stretch 41. Moreover, Cav1 gene expression and protein level increase during IVD degeneration, and high expression of Cav1 can be detected in IVD cells treated with interleukin-1β (IL-1β) to induce inflammatory responses 42. Therefore, elucidating the roles of Cav1, integrin β1 and NF-κB in mechanotransduction in degenerative progression induced by mechanical loading and analysis of their relationship is important for DDD therapy.

In this study, we set out to explore the role of Cav1 in mechano-regulation of integrin β1 and NF-κB signaling pathway during AF degeneration. A loaded cell culture system was used to apply various levels of mechanical loading, represented by cyclic tensile strain (CTS), to AFCs treated with or without IL-1β. Then, we evaluated the ECM synthesis, proliferation, migration, and inflammatory gene expression of AFCs after above treatments. Subsequently, AFCs were transfected with Cav1 plasmids or siRNA to explore the signaling pathways underlying these effects. *In vivo* studies were also performed to evaluate the effects of moderate mechanical loading on degenerative discs. Our findings imply that targeting Cav1 and integrin β1-mediated inactivation of the NF-κB signaling pathway by appropriate mechanical stimulation may rescue the cellular inflammation. In addition, effective recovery of the degenerative disc by dynamic traction *in vivo* may predict a new strategy for the treatment of DDDs.

## Materials and Methods

### Isolation and culture of AFCs

All animal-related procedures followed the NIH Guide for the Care and Use of Laboratory Animals and were approved by the Institutional Animal Care and Use Committee of Soochow University. To isolate AFCs, 8-week-old male Sprague-Dawley rats were sacrificed, and lumbar and caudal IVDs were subsequently harvested under aseptic conditions. After removing the surrounding soft tissues including muscle, ligaments and NP tissue, the remaining AF tissues were washed 3 times with phosphate-buffered saline (PBS, HyClone, Logan, UT, USA), minced into small pieces, and then digested with 2 mg/mL Collagenase I and Collagenase II (Yeasen, Shanghai, China) for 4-6 h. The so-obtained cell suspension was centrifuged at 1200 rpm for 3 min, and then cultured with DMEM/F12 containing 10% fetal bovine serum (FBS, HyClone, Logan, UT, USA) and 1% penicillin/streptomycin (Gibco, Grand Island, NY, USA) in a humidified incubator at 37 °C with 5% CO_2_. AFCs at passage 2 were used for all experiments in this study.

### Cell culture under mechanical loading

A Loaded Cell Culture System (Celload-300, Suzhou Haomian Precision Technology Co., Ltd, Suzhou, China) that provides adjustable longitudinal cyclic stretching was used to apply mechanical stimulation to AFCs. AFCs were plated on fibronectin-coated silicon chambers made from polydimethylsiloxane (PDMS, Dow Corning, Midland, MI, USA) at an initial density of 3,000 cells/cm^2^. After 24 h, AFCs were subjected to cyclic stretching with tensile strains of 0%, 2%, 5% or 12%, respectively at 0.5 Hz for 24 h. AFCs subjected to static culture without loading were used as the control group (Ctrl). Cells were transfected with pEX4-Cav1 plasmids (Gene-Pharma, Shanghai, China) or Cav1-specific small interference RNA (Gene-Pharma, Shanghai, China) for 24 h to overexpress or inhibit Cav1, followed by IL-1β and CTS treatment. To induce inflammation, IL-1β (20 ng/mL, Peprotech, London, UK) was supplemented in the culture medium during the entire course of mechanical loading. All cell cultures were performed in a humidified incubator at 37 ℃ with 5% CO_2_.

### Cytoskeleton staining and cell orientation analysis

The cytoskeleton and orientation analysis of this part were performed according to the methods published earlier 43. In brief, the cells were stained with TRITC-phalloidin (1:300, Yeasen, Shanghai, China) and DAPI (1:1000, Invitrogen by Thermo Fisher Scientific, Eugene, OR, USA) to observe the morphological changes of the cells treated with 0%, 2%, 5% or 12% CTS, respectively. Cell orientation was analyzed with the Image J software (NIH, Bethesda, MD, USA) from the images of phalloidin staining.

### Cell proliferation assay

AFCs in all groups were cultured with or without loading for 24 h. AFCs were then collected and fixed in 70% ethanol at 4 °C for at least 1 h, then incubated with RNase A (1 mg/mL) at 37 °C for 30 min. Subsequently, cells were stained with propidium iodide (50 mg/mL PI, Becton-Dickinson, Shanghai, China) in PBS, and analyzed with flow cytometry analysis apparatus (Guava^®^ easyCyte, Merck Millipore, Germany).

### Cell migration assay

AFCs were seeded on fibronectin-coated PDMS chambers. Upon reaching confluence, the monolayer was scraped with an aseptic 100 μL tip to create a wound. Subsequently, the medium was changed to the serum-free one and the champers were stretched with CTS of different magnitudes. The cell stretch system was placed on the cell incubator with a constant of 37 °C. Cells were photographed after wounding at 0 h, 12 h and 24 h, respectively, with a CCD camera attached to the microscope (Leica DMIRB, Weztlar, Germany) at ×5 magnification. The wound closure distances were determined using the microscope and the area of closure between the 0 h and 12 h (or 24 h) time points were calculated.

### RNA isolation and qPCR analysis

TRIzol reagent (Invitrogen, Carlsbad, CA, USA) was used to extract total RNA from AFCs under different conditions. The RNA concentration was measured using the NanoDrop 2000 spectrophotometers (Thermo Fisher Scientific, Waltham, MA, USA), and then 1 µg RNA was reverse-transcribed into cDNA using 5X All-In-One RT MasterMix (abm, Vancouver, BC, Canada) according to the manufacturer's instructions. qPCR was performed using the iQ SYBR Green Supermix (Bio-Rad, Hercules, CA, USA). Primer sequences (Sangon Biotech, Shanghai, China) of the genes used in this study were listed in Table 1. Relative mRNA expression of each gene was normalized to the housekeeping gene *Gapdh* and analyzed using the 2^-ΔΔCt^ method.

### Transcriptome sequencing and analysis

For transcriptome sequencing, total RNA was extracted from the cell samples subjected to 0%, 5% and 12% CTS, respectively, for 24 h using TRIzol reagent according to the manufacturer's protocol. The samples were then subjected to commercial RNA-Seq analysis (Novogene Sequencing Company, Beijing, China). Sequencing libraries were generated using NEBNext^®^ Ulta^TM^ RNA Library Prep Kit for Illumina^®^ (New England Biolabs, MA, USA) following the manufacturer's recommendations. Differential expression analysis of the three groups was performed using the DEGSeq R package (version 1.20.0). The original *p* values were adjusted using the Benjamini & Hochberg method. The corrected *p* value of 0.005 and log_2_ (Fold change) of 2 were set as thresholds for a significantly differential expression. Gene Ontology (GO) and Kyoto Encyclopedia of Genes and GENOMES (KEGG) pathway analyses were performed using the Metascape. The hierarchical clustering heat map was generated with the ggplot library.

### Cell transfection

Plasmids of pEX4-Vector, pEX4-Cav1, pcDNA3.1-Vector and pcDNA3.1-Integrin β1 (Gene-Pharma, Shanghai, China) were transiently transfected into AFCs at 70% confluence using Lipofectamine 2000 DNA Transfection Reagent (Invitrogen, California, USA) as suggested by the manufacturer. The small interfering RNA (siRNA) control (siCtrl) and Cav1siRNA (siCav1) were designed and synthesized by Gene-Pharma. The sequences of siCav1 oligos were 5'-GUAAAUACGUAGACUCCGATT-3'. And the control siRNA was also successfully constructed. The sequences of control siRNA oligos were 5'-UUCUCCGAACGUGUCACGUTT-3'. For transient transfection, siCtrl and siCav1 were carried out using siLentFect^TM^ Lipid Reagent (Bio-Rad, Hercules, CA, USA) following the manufacturer's protocol. After transfection, cells were stretched or stained for subsequent experiments.

### Western blot analysis

Total cellular proteins were extracted from AFCs at the end of the mechanical loading period using Tissue or Cell Total Protein Extraction Kit (Sangon Biotech, Shanghai, China) according to the manufacturer's protocol. These proteins were quantified using a BCA protein assay kit (Beyotime, Shanghai, China). Extracted proteins were denatured and separated in a 10% sodium dodecyl sulfate polyacrylamide gel electrophoresis (SDS-PAGE) gel (Beyotime, Shanghai, China), and then transferred by electrophoresis onto polyvinylidene fluroride (PVDF) membranes (Beyotime, Shanghai, China). Membranes were blocked in blocking buffer for 60 min and incubated with properly diluted primary antibodies at 4 °C overnight (All antibodies used for Western blot analysis were diluted as 1:1000). The membranes were washed and then incubated with respective horseradish peroxidase-conjugated secondary antibodies at room temperature for 1 h. Proteins were detected by autoradiography (Bio-Rad, Hercules, CA, USA) and the grayscale value was quantified using ImageJ software (NIH, Bethesda, MD, USA). The primary antibodies used in this study were listed in Table 2.

### Co-immunoprecipitation

AFCs were seeded at a density of 2.5 × 10^6^ into 10 cm plates for 24 h and then transfected with the plasmids of pEX4-Cav1 or pcDNA3.1-Integrin β1 for 48 h before harvest. Afterward, cells were rinsed twice with PBS, pelleted, and resuspended using modified radioimmunoprecipitation (RIPA) buffer (Upstate, VA, USA) supplemented with Complete Protease Inhibitor Cocktail (Roche Applied Science, IN, USA) for 1 h at 4 °C. Insoluble material was removed by centrifugation at 12000 rpm for 15 min at 4 °C. A small amount of lysate was used for Western blot analysis, and the remaining extracts were introduced 30 μL Agarose A+G (Santa Cruz Biotechnology, CA, USA) per sample for pre-clearing, at 4 °C for 2 h, and then centrifuged at 3000 rpm, at 4 °C for 5 min. The supernatants were incubated with 1 μg of anti-Cav1 (Cell Signaling Technology, MA, USA), anti-integrin β1 (Novus Biologicals, CO, USA) or anti-IgG (Cell Signaling Technology, MA, USA) antibody overnight at 4 °C. Then the antibodies were pulled down with 30 μL Agarose A+G at 4 °C for a further 2 h and centrifuged at 3000 rpm, at 4 °C for 5 min. The supernatant was discarded and the remaining sediment was rinsed with 500 μL washing buffer three times. After centrifugation, the sediment was added with 30 μL 2 × Sample Buffer and heated at 100 °C for 10 min. The bound complexes were separated using 10% SDS-PAGE and transferred onto PVDF membranes.

### Immunofluorescence

AFCs were fixed in 4% paraformaldehyde for 15 min and then permeabilized with 0.3% Triton X-100 in PBS for 10 min. Non-specific binding was blocked by 4% bovine serum albumin (BSA) at room temperature for 2 h. After incubation with anti-Cav1 (1:200, Abcam, Cambridge, UK ), anti-integrin β1 (1:500, Novus Biologicals, CO, USA) or anti-p65 (1:200, Abcam, Cambridge, UK) antibodies at 4 °C overnight, appropriate Alexa Fluor 488 second antibodies (1:1000, Abcam Cambridge, UK) were used for fluorescent labeling. Nuclei were stained with DAPI. Images were acquired using a fluorescence microscope (Carl Zeiss Microscopy, Thornwood, NY).

### Immunocytochemistry

Following CTS for 24 h, AFCs were rinsed with PBS twice and fixed at room temperature for 15 min in 4% paraformaldehyde. After 2 h of blocking in 10% donkey serum with 0.3% Triton X-100 and 1% BSA, all the cells were incubated overnight at 4 °C with anti-Collagen I (1:200, Abcam, Cambridge, UK), Collagen II (1:200, Novus Biologicals, CO, USA) and Aggrecan (1:200, GeneTex, CA, USA) antibodies, respectively. Afterward, cells were incubated with secondary antibody (HRP conjugated goat anti-Rabbit IgG, 1:100, Beyotime, Shanghai, China) for 2 h and developed with diaminobenzidine (DAB, Sangon Biotech, Shanghai, China) for 30 min and counterstained with hematoxylin. Evaluation of the immunocytochemistry was done by light microscopy (Carl Zeiss Microscopy, Thornwood, NY).

### Animal studies

Animal experiments were performed following the NIH Guide for the Care and Use of Laboratory Animals experiments were approved by the Institutional Animal Care and Use Committee of Soochow University. In this study, 3-month-old male Sprague-Dawley rats with an average body weight of 350 g were used. Rats were randomly divided into four groups. Sham group (caudal vertebrae were instrumented with K-wires only). Compression group (caudal vertebrae were immobilized using a custom-made external device to fix four vertebrae (Co7-10), and Co8-9 vertebrae underwent 2 weeks of compression to induce mild disc degeneration). Release group (Co8-9 vertebra underwent 2 weeks of compression to induce mild disc degeneration, followed by removing the external apparatus for 2 weeks). Traction group (Co8-9 vertebra underwent 2 weeks of compression to induce mild disc degeneration, followed by dynamic traction for 2 h every other day, for a period of 2 weeks) (Figure 8A). For the traction group, a custom-made caudal vertebrae traction system (Haomian Precision Technology Co, Ltd, Suzhou, China) that provides adjustable uniaxial cyclic stretching was used to apply moderate mechanical stimulation on the discs. A low frequency (0.1 Hz) with 40% body weight traction was applicable to mimic the lumbar traction therapy in the clinic.

### Histological staining

At the termination of the *vivo* experiments, rats were euthanized with 1.5% pentobarbital sodium (30 mg/kg rat body weight). The target discs of the caudal vertebrae were harvested for further analysis. Then the samples were fixed in 4% paraformaldehyde for 1 day and decalcified in 14% Ethylene Diamine Tetraacetic Acid (EDTA) for 30 d. Subsequently, discs were embedded in paraffin (Leica, Richmond, VA, USA), and cut into 5-μm sections in the coronal plane using a microtome (Leica, Heidelberg, Germany). Hematoxylin & Eosin (H&E) staining was performed to examine tissue histology. Briefly, the sections were stained with hematoxylin solution for 5 min and incubated in 1% acid ethanol (1% HCl in 70% ethanol). The sections were washed with pure water. Afterward, the sections were stained with an eosin solution for 3 min, dehydrated with graded alcohol and cleared in xylene. Safranin O-Fast green (S.O.) staining was performed to determine changes in proteoglycans. The sections were stained with a safranin O staining kit (Solarbio, Beijing, China) according to the recommended procedure from the manufacture.

### Immunohistochemistry

All the sections were blocked with 10% donkey serum with 0.3% Triton X-100 and 1% BSA for 2 h and incubated with anti-Cav1 (1:400, Cell Signaling Technology, MA, USA), anti-p-p65 (1:200, ABclonal, MA, USA), anti-COX-2 (1:200, HuaBio, Zhejiang, China), anti-Collagen I (1:200, Abcam, Cambridge, UK) and anti-IgG (1:400, Cell Signaling Technology, MA, USA) antibodies at 4 °C overnight, followed by incubation with secondary antibody (HRP conjugated goat anti-Rabbit IgG, 1:500, Beyotime, Shanghai, China) for 2 h. Sections were visualized with DAB (Sangon Biotech, Shanghai, China) for 30 min and counterstained with hematoxylin. Evaluation of the immunocytochemistry was done by light microscopy.

### Statistical analysis

All experiments were designed with 3 replicates and repeated at least 3 times. The total number of samples equals 9 times that of the number of total groups. Quantitative data were provided as the mean ± standard deviation (SD). Statistical analysis was performed using a one-way analysis of variance (ANOVA), followed by Tukey post hoc comparison (GraphPad Software 7.0, CA, USA). A difference is considered statistically significant if *p* is less than 0.05.

## Results

### The morphology, proliferation and migration of AFCs changed under different mechanical loading conditions

**Figure 1:**
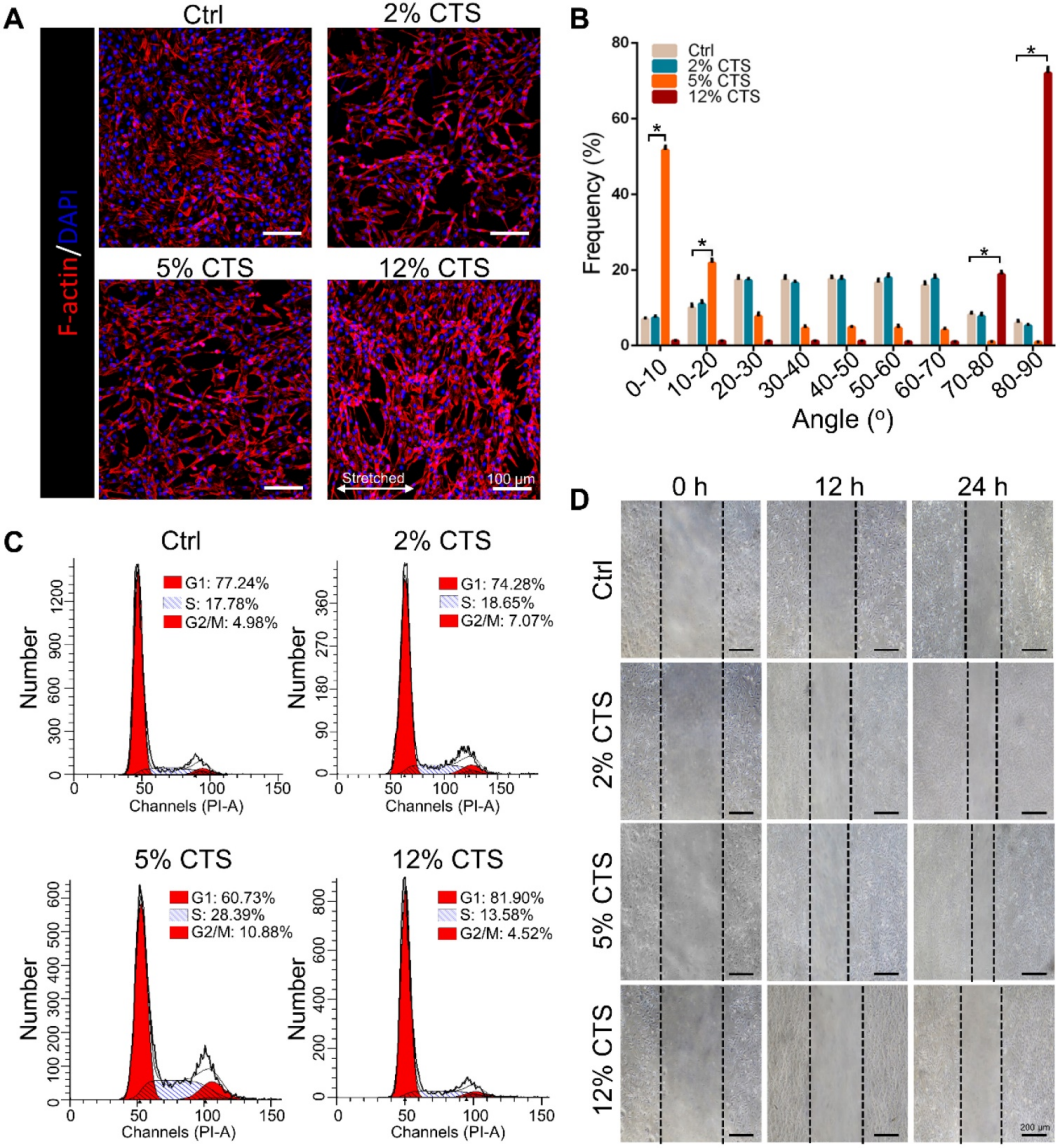
Effects of mechanical loading on the morphology, proliferation and migration of AFCs. AFCs were subjected to CTS at the magnitude of 0% (Ctrl), 2%, 5% and 12%, respectively, for 24 h. **(A)** Morphology of AFCs under different mechanical loading. Red indicates actin filaments and blue indicates nuclei.** (B)** Orientation analysis of AFCs under different mechanical loading conditions. **(C)** The cell cycle distribution of AFCs under different mechanical loading conditions. **(D)** The effect of mechanical stimulation on cell motility was examined using cell migration assay (*p<0.05 vs. Ctrl). The error bars indicate SD. N = 3.

To evaluate the effects of mechanical loading on AFC behavior, we treated AFCs with uniaxial stretching. Four different magnitudes, i.e., 0% (Ctrl), 2%, 5% and 12% CTS, respectively, were used for 24 h. AFCs under static condition and 2% CTS showed flatted cell shapes and exhibited random direction. However, the morphology of stretch-treated cells became spindle-like when treated with 5% or 12% CTS. Cells in the 5% CTS group tended to spread along the loading direction, whereas cells treated with 12% CTS grew along the perpendicular direction of mechanical loading (Figure 1A, B). Besides, the percentage of cells in S phase significantly increased after treated with 5% CTS, indicating that 5% CTS yield a higher level of cell proliferation than other groups (Figure 1C, S1). The migration of AFCs under different loading conditions was evaluated using wound-healing assay. From the results, 5% CTS promoted AFC migration significantly (Figure 1D, S2). Based on the above results, a CTS of 5% could be considered as moderate mechanical stimulation, which might benefit cell growth. In comparison, 12% CTS had negative effects on cell growth, so it was considered as excessive mechanical loading.

### Moderate mechanical loading promoted the expression of ECM-related proteins in AFCs

**Figure 2:**
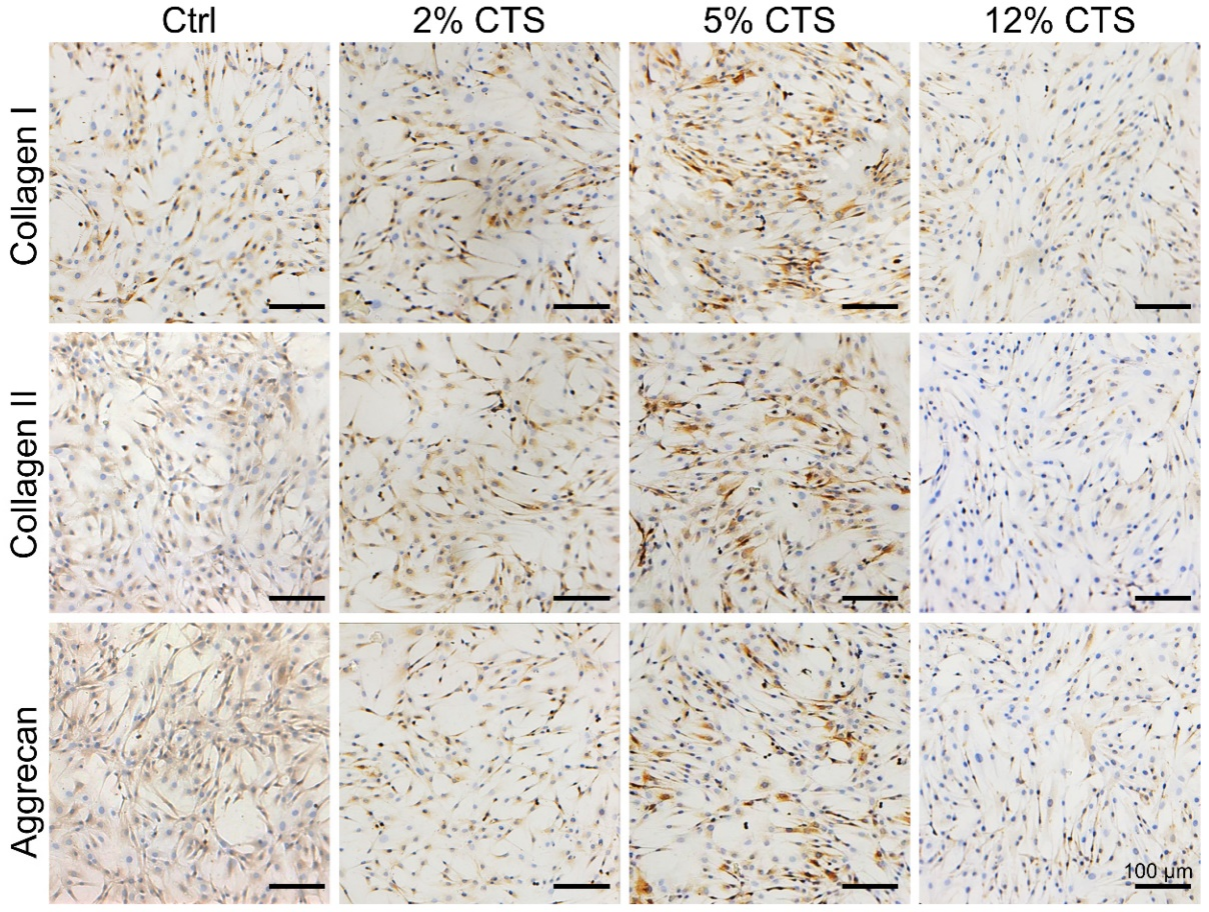
Effects of mechanical loading on the matrix anabolism of AFCs. Immunocytochemistry was performed to detect the expression level of anabolic matrices (Collagen I, Collagen II and Aggrecan) of AFCs under different mechanical loading conditions. N = 3. Scale bar = 100 µm.

Expressions of ECM-related proteins (Collagen I, Collagen II and Aggrecan) in AFCs were investigated by immunocytochemical staining. The expression levels of Collagen I, Collagen II and Aggrecan were higher in AFCs subjected to 5% CTS, compared to cells under static conditions and subjected to 2% or 12% CTS (Figure 2). Therefore, 5% was considered as a moderate mechanical magnitude of CTS controlling anabolism of AFCs. These results also indicate that moderate mechanical stimulation could promote the synthesis of ECM and had the potential to promote repair and regeneration of the tissue.

### The expression of pro-inflammatory genes in AFCs changed under different mechanical loading conditions with or without IL-1β treatment

**Figure 3:**
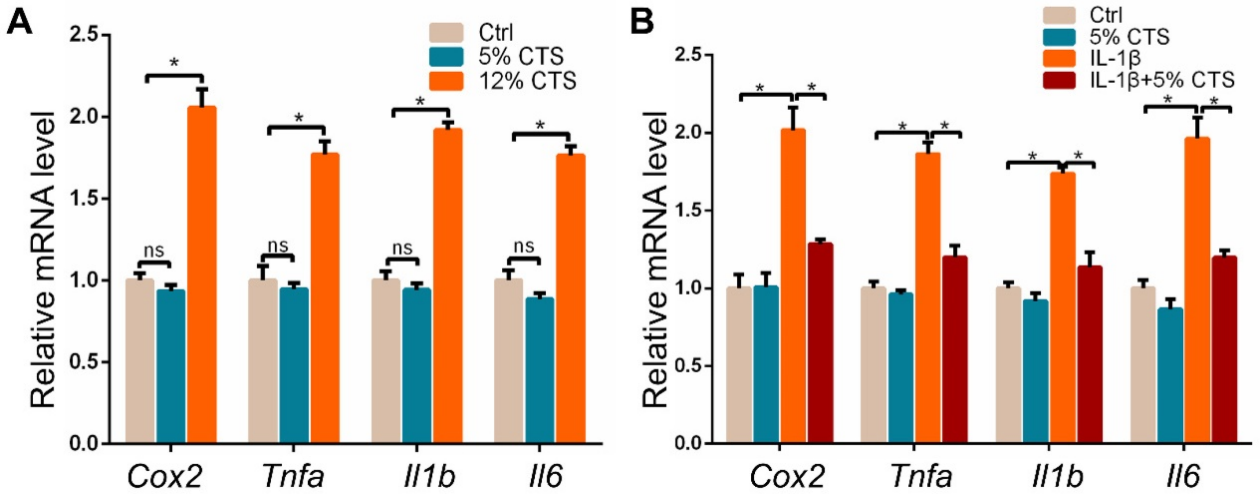
Effects of mechanical loading on the mRNA expression of pro-inflammatory genes in AFCs. **(A)** qPCR analyses of relative expression of pro-inflammatory genes (*Cox2*, *Tnfa*, *Il1b* and *Il6*) in AFCs under different mechanical conditions. **(B)** qPCR analyses of relative expression of pro-inflammatory genes (*Cox2*, *Tnfa*, *Il1b* and *Il6*) in AFCs treated with 5% CTS in the presence or absence of IL-1β. (*p<0.05, ^ns^*p*>0.05 vs. Ctrl). The error bars indicate SD. N = 3.

Given the effects of mechanical loading on the growth and metabolism of AFCs, we further explored the inflammatory responses of AFCs after 24 h of CTS by evaluating the expression of pro-inflammatory genes. The results showed that pro-inflammatory genes, including *Cox2*, *Tnfa*, *Il1b* and *Il6*, significantly increased in AFCs treated with 12% CTS. Nevertheless, 5% CTS had no obvious influence on the expression of pro-inflammatory genes in AFCs compared to those under static condition (Figure 3A). This may be due to the low expression of these pro-inflammatory genes in AFCs under static condition. Since 5% CTS was beneficial to cell growth, we speculated that mechanical stimulation of this level could execute anti-inflammatory effects on AFCs. Here, we treated AFCs with IL-1β to induce acute inflammatory response, followed with 5% CTS. The results indicated that 5% CTS was able to significantly reverse the elevated expression of pro-inflammatory genes in IL-1β treated AFCs (Figure 3B). Taken together, these results suggest that 5% CTS attenuated IL-1β-induced inflammatory responses, but 12% CTS exacerbated these processes.

### Transcriptome sequencing analysis of AFCs under different mechanical loading conditions

**Figure 4:**
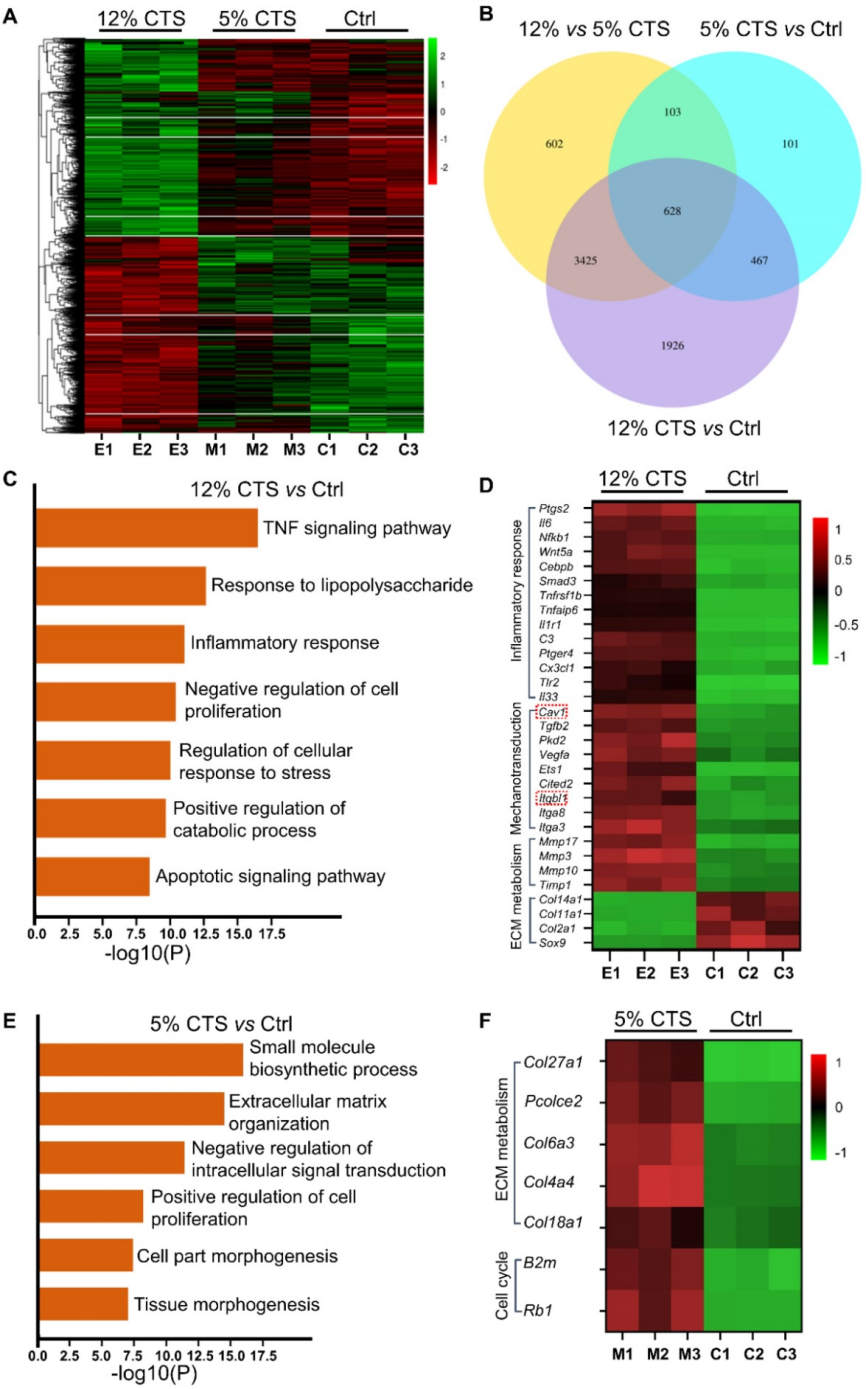
Gene expression profiles of AFCs under different mechanical loading conditions. AFCs were subjected to the CTS of 5% and 12% for 24 h and gene expression was analyzed by transcriptome sequencing. Cells under static condition served as the control. **(A)** Heatmap of the differentially expressed genes for cells as described above.** (B)** Venn diagram showed genes that were differentially expressed in AFCs cultured under 12% CTS, 5% CTS or static conditions. **(C)** The Gene Ontology (GO) and pathway enrichment analyses showed the top seventh biological processes activated by 12% CTS. **(D)** Heatmap showed the changes in the expression of pro-inflammatory genes, mechanosensitive genes and catabolic genes in AFCs of 12% CTS and Ctrl groups. **(E)** The Gene Ontology (GO) enrichment and pathway enrichment analyses showed the top sixth biological processes activated by 5% CTS. **(F)** Heatmap showed the changes in the expression of metabolic genes and proliferation-associated genes in AFCs of 5% CTS and the Ctrl groups. E, excessive; M, moderate; C, Ctrl. N = 3.

To investigate the transcriptomic changes of gene expression in AFCs treated with different mechanical loading, RNA sequencing was used to quantify mRNA levels in AFCs. The gene expression heatmap showed that the expression patterns were similar between the 5% CTS and Ctrl groups, while they were largely different from the 12% CTS group (Figure 4A). Venn diagram showed that there were 6646 and 4758 differentially expressed genes when comparing 12% CTS group with Ctrl and 5% CTS groups, respectively, while the number was only 1299 for 5% CTS and Ctrl group comparison (Figure 4B), indicating that AFCs changed dramatically under 12% CTS treatment. Afterward, we performed Gene Ontology (GO) and pathway enrichment analyses, and the top seven biological processes or pathways up-regulated by 12% CTS were shown in Figure 4C. Among them, inflammatory response, catabolic process, and apoptotic pathway were activated, while cell proliferation was negatively regulated in the 12% CTS group, which was consistent with our previous flow cytometry results (Figure 1C). Specifically, a significant upregulation of inflammatory genes (*Ptgs2, Il6, NFkB1, etc.*), mechanosensitive genes (*Cav1, Itgbl1, Itga3, etc.*) and catabolic genes (*Mmp17, Mmp3, Timp1, etc*) were found in the cells treated with 12% CTS, and the anabolic genes (*Col2a1, Col11a1, Col14a1, etc.*) were down-regulated (Figure 4D). On the contrary, small molecule biosynthetic process, ECM organization and cell proliferation were up-regulated in the 5% CTS group compared with the Ctrl group (Figure 4E). The heatmap revealed an upregulation of anabolic genes (*Col27a1, Col6a3, Col4a4, etc.*) in cells treated with 5% CTS (Figure 4F), indicating that 5% CTS was beneficial to AFCs.

### The expression of Cav1 and integrin β1 in AFCs changed under different mechanical loading conditions

**Figure 5:**
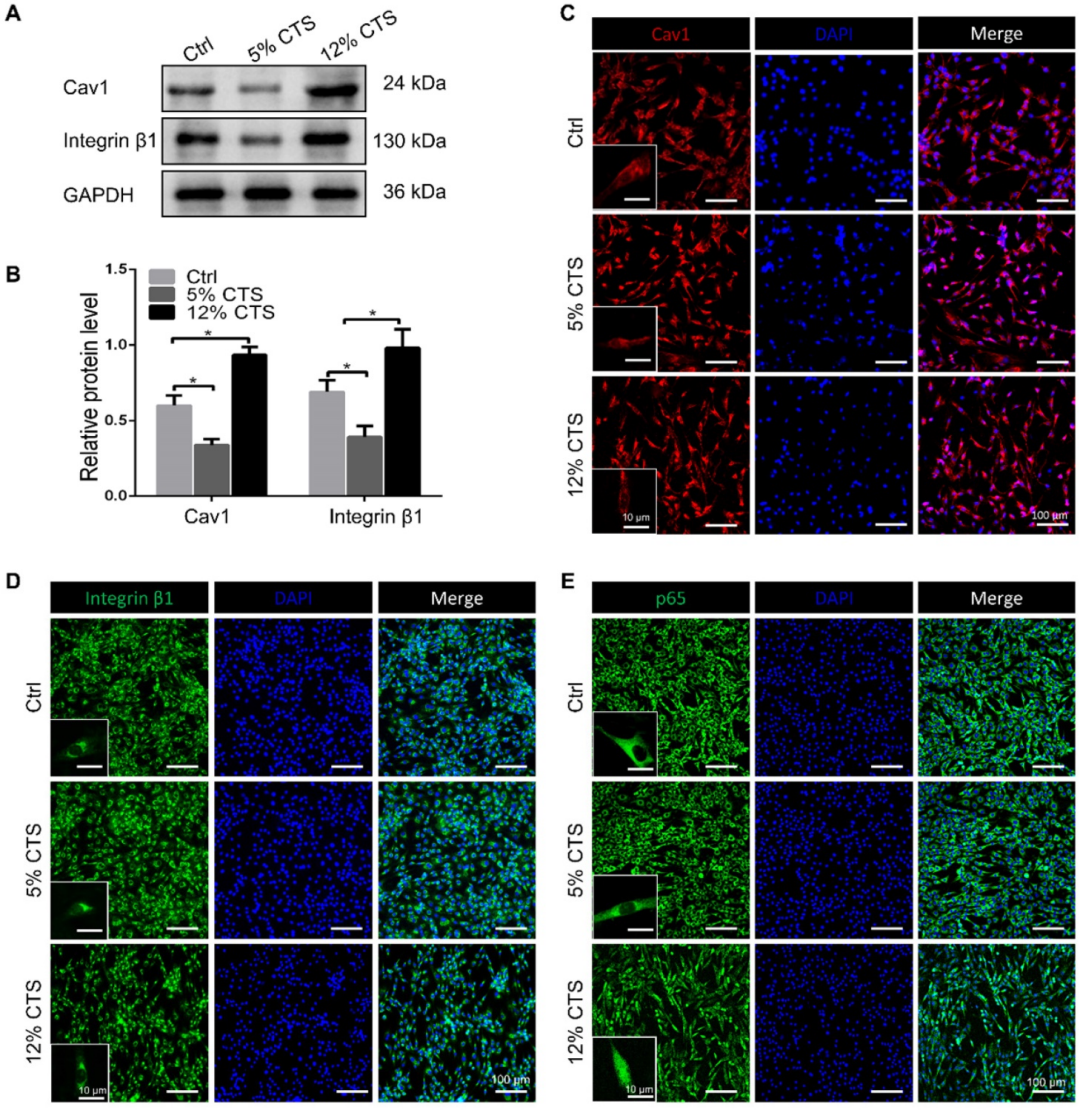
Change of expression of Cav1 and integrin β1 in CTS treated AFCs. **(A, B)** Change of protein expression of Cav1 and integrin β1 in AFCs under different mechanical conditions. **(C-E)** Location of Cav1, integrin β1 and p65 in AFCs under different mechanical conditions. (*p<0.05 vs. Ctrl). The error bars indicate SD. N = 3.

To elucidate the mechanisms of mechanical signal transduction underlying CTS-induced inflammatory response, the levels of two mechanosensitive proteins, Cav1 and integrin β1, were directly cheeked after CTS treatment. Clearly, the expression of Cav1 and integrin β1 was down-regulated in the 5% CTS group, which might result in the decrease of cellular sensitivity to physical signals. In contrast, 12% CTS significantly increased the expression of these two proteins (Figure 5A, B). The cellular localization of Cav1 protein expression was detected by immunofluorescence. Interestingly, Cav1 was presented at the plasma and membrane of AFCs, and no further increase of nuclear Cav1 was measured upon CTS of 5% or 12% (Figure 5C). Similarly, integrin β1 was also located at the plasma and membrane under both static and mechanical conditions (Figure 5D). Now that these two mechanosensitive proteins were always presented at the plasma and membrane of AFCs under any mechanical conditions, they might transduce the external mechanical cues into specific biological signals through the intracellular signaling pathway downstream. p65 nucleus translocation plays an important role in modulating the intracellular pro-inflammatory response. According to the immunofluorescence results, p65 was located in the cytoplasm under static and 5% CTS conditions, but was rapidly translocated into the nucleus upon 12% CTS (Figure 5E). These data suggest that the two proteins might participate in CTS-induced AFC inflammatory responses, and their functions might rely on the nucleus translocation of p65.

### Cav1-mediated signaling pathway was involved in CTS-induced inflammatory responses of AFCs

**Figure 6:**
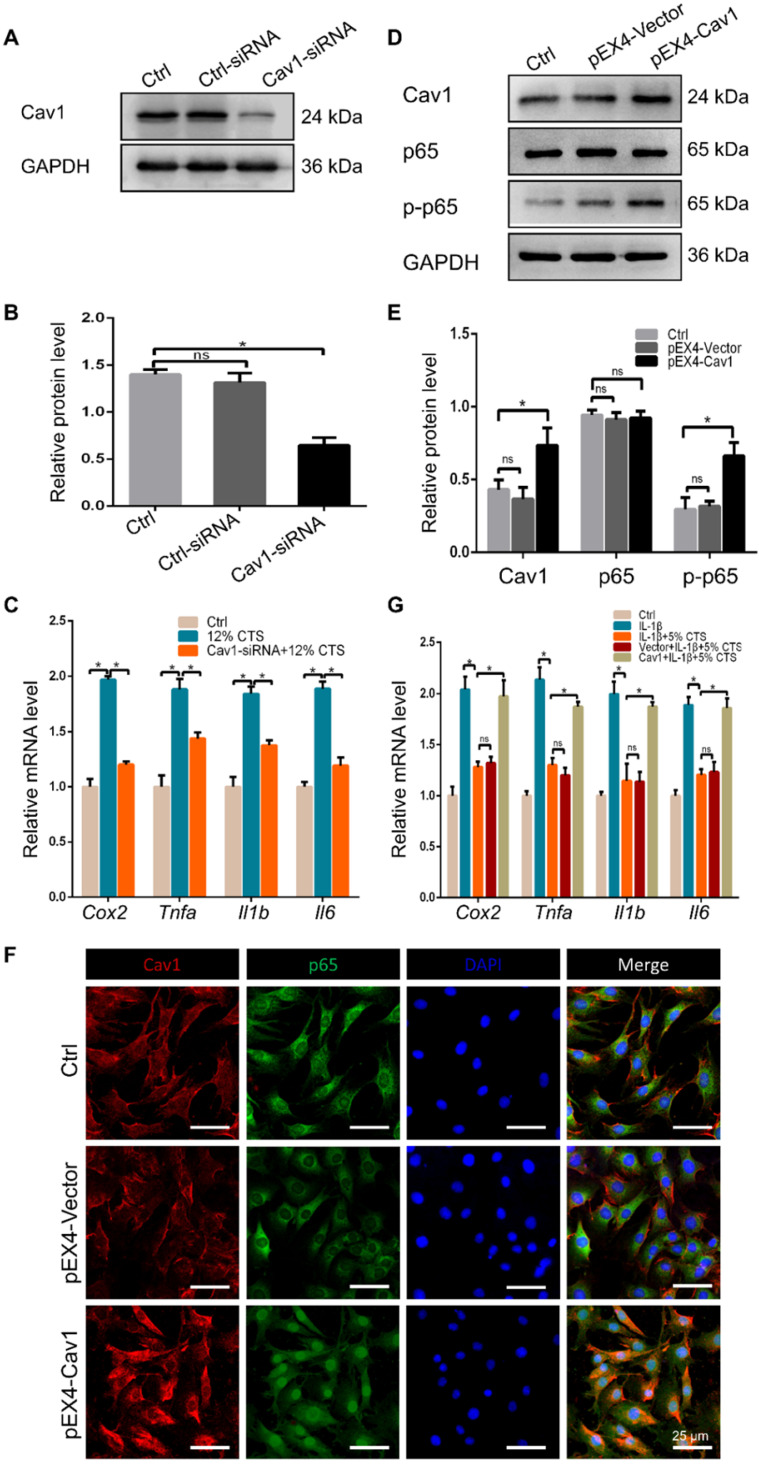
Cav1-mediated signaling pathway was involved in CTS-induced inflammatory responses of AFCs. **(A, B)** Efficiency of Cav1-specific siRNA in AFCs. **(C)** qPCR analyses of relative expression of pro-inflammatory genes (*Cox2*, *Tnfa*, *Il1b* and *Il6*) in AFCs subjected to 12% CTS and transfected with or without Cav1-siRNA.** (D, E)** Protein level of p-p65 changed in AFCs transfected with pEX4-Cav1 plasmids. **(F)** Immunofluorescence images of Cav1 (red), p65 (green) and DAPI (blue) in AFCs transfected with pEX4-Vector or pEX4-Cav1 plasmids. **(G)** qPCR analyses of relative expression of pro-inflammatory genes (*Cox2*, *Tnfa*, *Il1b* and *Il6*) in AFCs of Ctrl, IL-1β, IL-1β+5% CTS, Vector+IL-1β+5% CTS and Cav1+ IL-1β+5% CTS groups, respectively. The error bars indicate SD. N = 3.

To identify the involvement of Cav1 in excessive CTS-induced inflammation, small interfering RNA (siRNA) duplexes were used to inhibit Cav1 in AFCs. Successful suppression of Cav1 was assessed by Western blot analysis (Figure 6A, B). And knockdown of Cav1 in AFCs treated with 12% CTS led to a reduction of pro-inflammatory gene level (Figure 6C). Subsequently, pEX4-Cav1 plasmids were introduced into AFCs and utilized to overexpress Cav1. As shown, Cav1 over-expression in AFCs increased the protein level of the phosphorylation of p65 (p-p65), which is an indicator of p65 activation and acts as the major transcription factor of the NF-κB signaling pathway (Figure 6D, E). Immunofluorescence results further confirmed that p65 rapidly accumulated in the nucleus in response to Cav1 overexpression (Figure 6F). To further confirmed the role of Cav1 in moderate CTS-exhibited anti-inflammatory effects, acute inflammation was induced in AFCs by adding IL-1β directly to the culture medium (20 ng/mL) before applying CTS. Subsequently, pEX4-Cav1 plasmids were transfected into the cells. RT-qPCR data showed that overexpression of Cav1 abrogated the anti-inflammatory effects induces by 5% CTS and resulted in up-regulation of pro-inflammatory genes (Figure 6G). These data indicate that Cav1 might act as a crucial mechanosensitive factor, which regulated the inflammatory response in CTS-treated AFCs with the regulation of p65 nucleus translocation and downstream inflammatory signals.

### Cav1 interacted with integrin β1 directly

**Figure 7:**
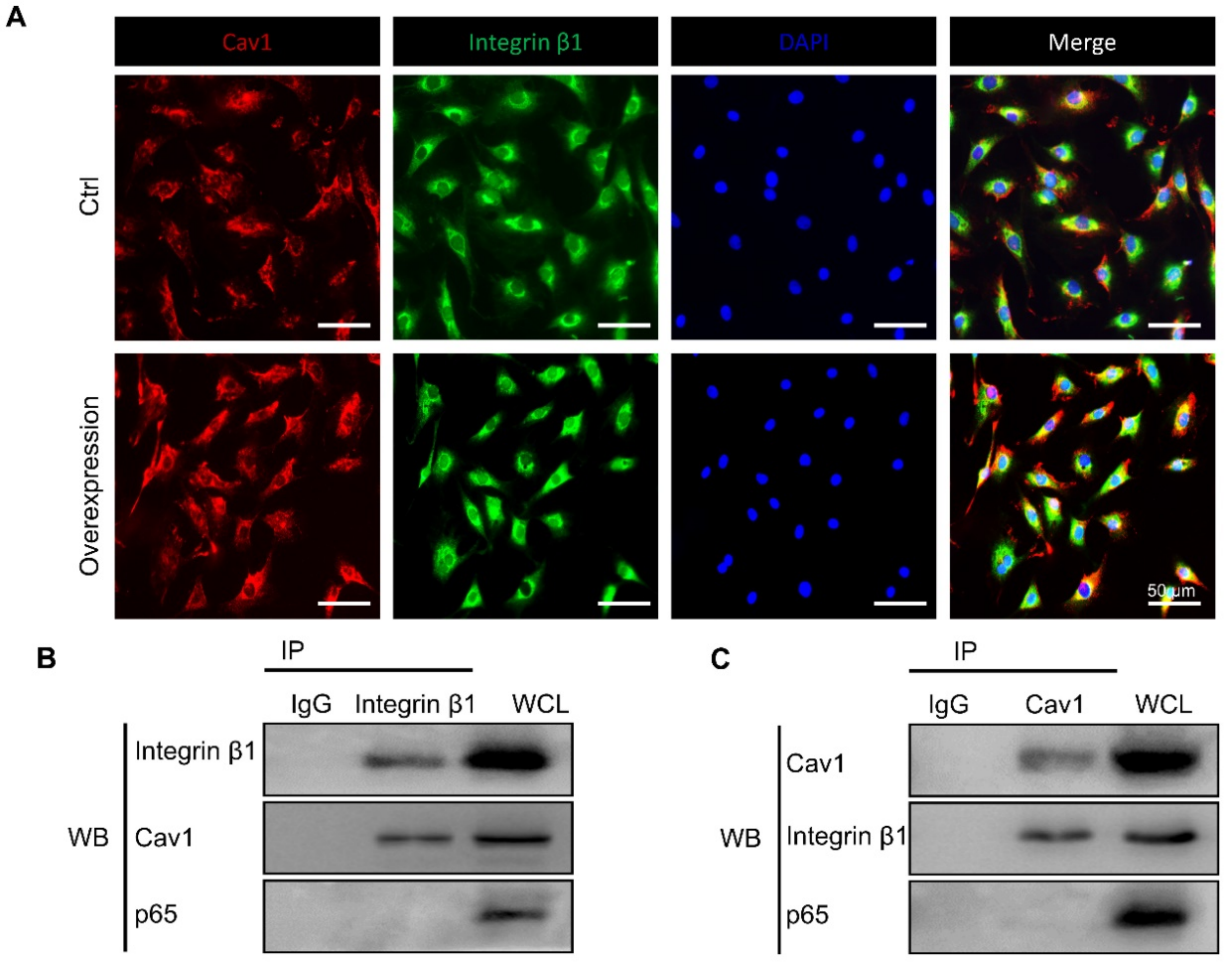
The interaction between Cav1 and integrin β1. **(A)** Immunofluorescence images of Cav1 (red), integrin β1 (green) and DAPI (blue) in AFCs transfected with (Group of Overexpression) or without (Group of Ctrl) pEX4-Cav1 and pcDNA3.1-Integrin β1 plasmids. Co-IP assays were performed in AFCs transfected with pEX4-Cav1 **(B)** or pcDNA3.1-Integrin β1 **(C)** plasmids. Cell lysates were immunoprecipitated with either IgG, anti-Cav1 or anti-integrin β1 antibody and immunoblotted with indicated antibodies. While whole cell lysates (WCL) were immunoblotted with indicated antibodies. N = 3. Scale bar = 50 µm.

Previous studies have shown the reciprocal regulation between Cav1 and integrin β1 which relied on varying matrix stiffness 44. Based on the above results, we hypothesized that Cav1 might connect integrin β1 during the inflammatory response induced by external mechanical cues. To verify our hypothesis, we first applied immunofluorescent staining to trace the location of the two proteins in AFCs transfected with or without pEX4-Cav1 and pcDNA3.1-Integrin β1 plasmids. In this experiment, Cav1 and integrin β1 co-localized in the plasms of AFCs (Figure 7A). We then transfected AFCs with pEX4-Cav1 or pcDNA3.1-Integrin β1 plasmids to induce Cav1 or integrin β1 overexpression. Western blot assays showed significant up-regulation of integrin β1 (Figure S3A, B) or Cav1 (Figure S3C, D) protein level in AFCs transfected with pEX4-Cav1 or pcDNA3.1-Integrin β1 plasmids, respectively. Based on the results above, we predicted there is certain relationship between these two mechanosensitive proteins.

To figure out whether Cav1 and integrin β1 interacted with each other directly, we transfected AFCs with pEX4-Cav1 or pcDNA3.1-Integrin β1 plasmids and then conducted co-immunoprecipitation (Co-IP) to verify their relationship. Indeed, integrin β1 was precipitated with an anti-Cav1 antibody (Figure 7B), while Cav1 could also be precipitated with an anti-integrin β1 antibody (Figure 7C). However, p65 could not be precipitated with either Cav1 or integrin β1 antibody, which indicated that p65 nucleus translocation was indirectly regulated by the above mechanosensitive proteins. These results support that Cav1 and integrin β1 could interact with each other directly, and these two proteins might play synergistic roles during the inflammatory response induced by external mechanical cues.

### Moderate mechanical stimulation promoted recovery of degenerative disc *in vivo*

**Figure 8:**
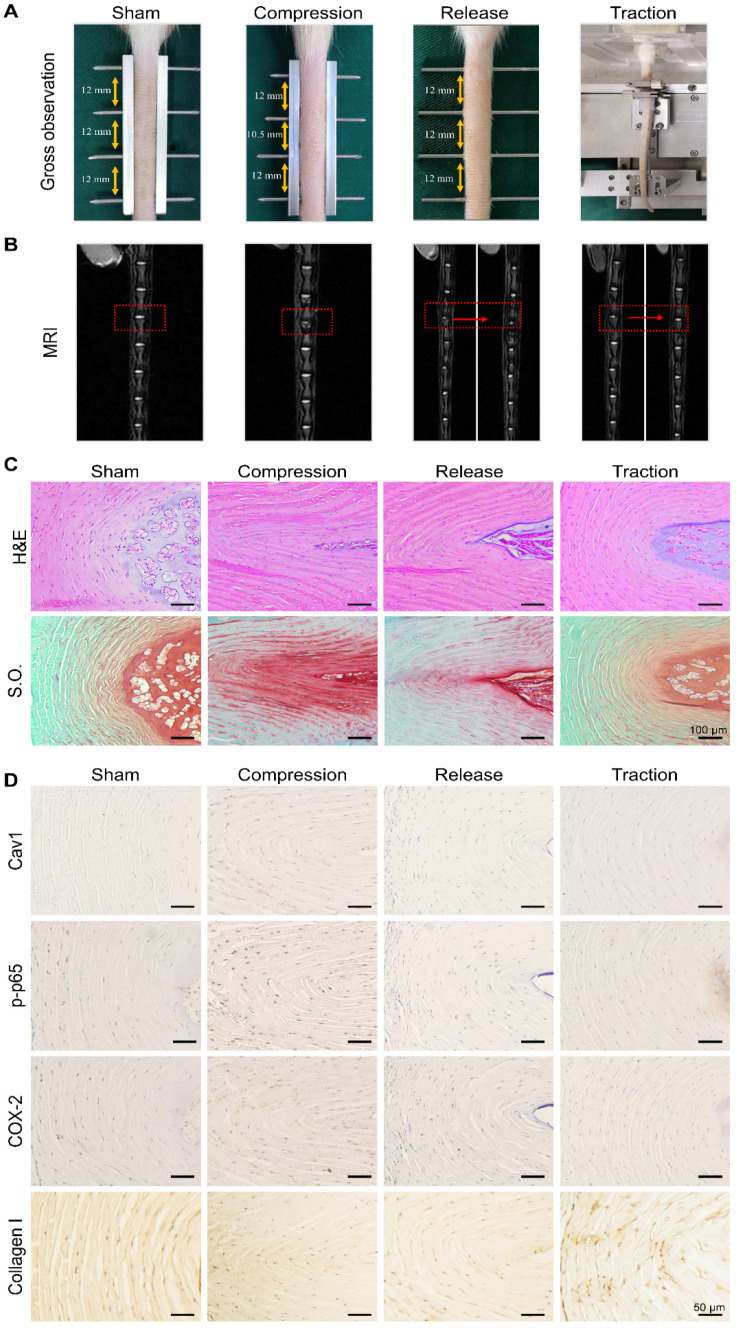
** Effects of moderate mechanical stimulation on disc repair and regeneration *in vivo***.** (A)** Gross observation of IVDs in the groups of sham, compression, release and traction. Magnetic resonance images **(B)**, H&E and Safranin O (S.O.) staining **(C)** of IVDs from the groups as described above. **(D)** Immunohistochemical expression of Cav1, p-p65, COX-2 and Collagen I in IVDs from the groups as described above. Negative control was shown in Figure S4. N = 3.

To further examine the feasibility of restoring degenerative discs by means of dynamic traction, the caudal vertebrae of rats were first compressed for 2 weeks to induce mild disc degeneration and then treated with moderate mechanical stimulation for 2 h every other day, for a period of 2 weeks (Figure 8A). After 2 weeks of compression, magnetic resonance images revealed a decrease in disc height and NP water content when compared to the sham group (Figure 8B). Histological examinations were also performed to analyze the morphologic changes of the discs in each group. In both H&E and S.O. staining of disc sections, the collagen fibers of the AF arranged wavily and more chondrocyte-like cells appeared in the AF region (Figure 8C). Following moderate mechanical stimulation for 2 weeks, magnetic resonance images showed the recovery of NP water content (Figure 8B), and H&E and S.O. staining results showed typical morphologic recovery of the discs after dynamic traction (Figure 8C). In general, the degenerative discs after traction showed better restoration when compared to that of rats in the release group. Furthermore, we performed the immunohistochemical staining assay to analyze the expression of Cav1, p-p65, COX-2 and Collagen I in the AF of each disc from different groups. The data demonstrated that the expression of Cav1, p-p65 and COX-2 upregulated in the compressed discs, but reduced significantly in the discs treated with moderate mechanical stimulation (Figure 8D). However, the expression of Collagen I showed a reverse trend with a high expression level in the discs treated with moderate mechanical stimulation, representing a kind of compensatory repair of degenerative discs (Figure 8D). Consistent with *in vitro* results, moderate mechanical stimulation may have beneficial effects for the repair of degenerative discs, which may rely on the Cav1-mediated signaling pathways.

## Discussion

In this study, we demonstrated that moderate mechanical loading relieved cellular inflammation induced by IL-1β via inhibiting the Cav1-mediated integrin β1 and NF-κB signaling pathway. IVD is a pad of fibrocartilage that transmits loads and provides flexibility to the spine 45. Therefore, IVD is considered to be situated in a loaded environment, in which the cells are subjected to mechanical stimuli, involving in tensile, compressive and shear stresses 18. Recent studies suggested the possibility of aberrant mechanical loading stresses in the aetiology of disc degeneration 46, 47. It is reported that excessive mechanical loading may result in deleterious changes of the discs by decreasing the expression level of anabolic genes, while simultaneously accelerating the expression of catabolic genes, such as *Mmps* and *Adamts* 48. In contrast, moderate mechanical loading is important for a healthy IVD by promoting the anabolism of ECM and contributing to the regeneration of the tissue 49. It is known that cells embedded in the discs can sense various mechanical signals, originated from the extracellular microenvironment, and transduce them into intracellular biochemical signals to influence cell fates. Moreover, recent studies have elucidated the mechanosensitive mechanisms of tissue (re)modeling 50-52. For instance, mechanical loading at the physiological level improves the proliferation, migration, differentiation of cells, which finally contributes to tissue remodeling 53, 54. Here, we used a loaded cell culture system which applied mechanical loading to cells* in vitro* to partially mimic the *in vivo* mechanical environment of AFCs. Of note, 12% CTS led to inflammatory responses in AFCs, while 5% CTS promoted cell migration, proliferation and ECM synthesis and showed protection from cellular inflammation. Furthermore, both *in vitro* and *in vivo* studies implied that decreased Cav1 expression could be a critical factor in anti-inflammatory effects and disc repair. Overall, these findings provide new insights into the novel mechanism underlying the repair and regeneration of degenerative disc induced by physiologic stimulation.

Mechanical stimulation underlies a very large coterie of cell behaviors, including cell morphology, proliferation, motility, gene expression, enzyme and matrix production 55. Here, we found that AFCs, under static condition, showed a flattened cell shape, but the morphology of stretch-treated cells became slender and spindle-like. We also found that AFCs in the 12% CTS group grew along the perpendicular direction of longitudinal mechanical force, in contrast to random directions of cells in the control group and 2% CTS group. (Figure 1A, B). Such a difference in cell morphology and alignment might attribute to the cytoskeletal remodeling 56. However, the explicit mechanism underlying these biological processes remains to be further elucidated. Previous studies have demonstrated that the proliferation, migration and ECM synthesis of the native cells are important biological processes underlying tissue repair 57-59. In addition, certain mechanical stimulation can promote cell proliferation, increase ECM production, and benefit for tissue repair 60, 61. Moreover, mechanical stimulus is reported to achieve increased IVD cell proliferation and ECM deposition 62-64. In our study, we found that 5% CTS promoted AFC proliferation, migration and ECM synthesis, but 12% CTS had the opposite effects (Figure 1C-D and Figure 2) and increased the expression of inflammatory genes, i.e., *Cox2*, *Tnfa*, *Il1b*, etc. (Figure 3A). Combined with the results of transcriptome sequencing analysis, 12% CTS could activate the inflammatory response, while 5% CTS increased the expression of ECM-related genes (Figure 4). Thus, 5% CTS was defined as moderate mechanical stimulation, which benefited cell growth. In contrast, 12% CTS was considered as excessive mechanical stimulation that could lead to inflammatory responses.

Inflammation in response to mechanical loading has been investigated in various studies. For instance, exposure of chondrocytes to excessive mechanical loading resulted in upregulation of inflammatory cytokines and matrix proteinases, such as IL-1β, TNF-α, MMP-3 and MMP-13 65, 66. Naoto and colleagues further explored the mechanisms underlying the mechanics-induced inflammatory response and demonstrated that excessive mechanical loading increased the sensibility of mechanosensors, such as integrin and focal adhesion kinase (FAK) 67. In contrast, moderate mechanical loading has protective effects on inflammatory responses in chondrocytes. Thompson et al. demonstrated that the production levels of NO and PGE_2_ in articular chondrocytes induced by IL-1β were declined after subjected to CTS at 0.33 Hz, 10% strain 68. Guilak et al. concluded that moderate loading was necessary for articular cartilage repair by modulating joint inflammation 69. Lee et al. reported that mechanical loading could recover the injured tendon through the regulation of macrophage polarization 70. Taken together, these findings point out that moderate mechanical stimulation can recover the injured tissues by reducing local inflammation. Recently, there is increasing evidence indicating that mechanical traction is one of the most effective physical therapy for alleviating symptomatic pain of IVD 71. In a study, moderate CTS was reported to decrease the expression of catabolic genes in the AF under inflammatory conditions 72. As a result, mechanical loading was proposed as a therapeutic option for the repair of IVD. In this study, we observed a decrease in pro-inflammatory cytokines in IL-1β-treated AFCs after subjected to 5% CTS (Figure 3B). However, how IVD cells sense a mechanical stimulation and convert it into a biochemical signal to regulate inflammatory response in AFCs remains elusive.

Integrin, focal adhesion kinase (FAK) and cadherin are known as mechanosensitive molecules that sense mechanical loads in cells 73. Integrin is a transmembrane protein which acts as a receptor that binds to the ligands of ECM and cell-surface, while cadherin, a calcium-dependent cell adhesion protein, mediates homotypic adhesion between cells. Usually, cadherin functions as an upstream regulator of integrin activation and localization. For instance, Schwartz et al. demonstrated that the intercellular mechanosensitive complex of PECAM1, VEGF receptor and cadherin could transmit external force, activate integrins and finally lead to cellular alignment in response to shear stress 74. Indeed, integrins and cadherins have been historically regarded as functionally and spatially crosstalk receptors, which determine the organization of mechanical signals at both cell and tissue level 75, 76. However, the assembly of integrin induced by external force also relies on another mechanosensitive protein of Cav1, which is the core structural protein of caveolae 77. The mechanical forces, such as shear stress, pressure and stretch, are all shown to have various effects on caveolae. The absence of caveolae can make the vasodilator generating pathways lose their function in response to shear stress 78. Caveolae deform under physiological mechanical stretch, but return to their original shape once the stimulus is removed 79. Cav1 is critical for the pressure-induced myogenic tone, which has a major role in the regulation of blood flow 80. According to recent studies, Cav1 is also reported to be involved in mechanical loading-related disc degeneration and repair 81. In this study, we found that the expression of Cav1 and integrin β1 increased in AFCs treated with 12% CTS (Figure 4D and 5A, B), while significantly decreased after treated with 5% CTS (Figure 5A, B). Moreover, Cav1 and integrin β1 interacted with each other directly (Figure 7) and overexpression of Cav1 activated the NF-κB signaling pathway (Figure 6D-F), which plays a critical role in cellular inflammatory responses under mechanical loading 82. However, the relationship between Cav1 and mechanical loading-induced disc degeneration or repair needs further investigation.

Disc degeneration includes marked loss of water content in the NP, random and disorganized arrangement of the fibers and the changes of collagen content. It was previously demonstrated that the height of disc and water content of NP can be recovered by traction. Kroeber et al. used a rabbit model to investigate the regeneration of the disc by dynamic disc distraction for 28 days 23. Luo et al. revealed that the degenerative discs of the rat caudal vertebrae could be repaired by immobilization-traction for 8 weeks 83, 84. However, it is inconvenient for the patients to have the lumbar traction for such a long period of time. Thus, more effective and convenient treatments are necessary. In this study, we first established an early degenerative disc model by compressing the rat tail vertebrae for 2 weeks and then treated the disc with timely and controllable dynamic traction in an attempt to reverse the structure and function of the discs (Figure 8A). Clearly, after 2 weeks of dynamic traction, the water content of NP largely recovered and the compressed AF became more organized (Figure 8B, C). Meanwhile, the elevated expression of COX-2, Cav1 and its down-stream p-p65 in the degenerative discs was restrained after applying dynamic traction. Together with results from *in vitro* studies, we speculate that Cav1-mediated p65 activation might be a critical signal in the mechanotransduction of the disc and is likely involved in the overloading-induced degeneration of disc. Nonetheless, further studies are needed to explore the crosstalk between Cav1 and p65 signaling pathways* in vivo*, and functional evaluation of recovered IVDs using biomechanical tests is necessary.

## Conclusions

In summary, in this study we investigated the effects of mechanical loading on discs at the cellular and tissue levels through *in vitro* and *in vivo* models. We found that excessive mechanical loading (12% CTS) suppressed AFC proliferation and migration, and increased the expression levels of inflammatory genes. On the contrary, moderate mechanical loading (5% CTS) rescued the inflammatory responses and enhanced AFC proliferation, migration and ECM synthesis by suppressing Cav1-mediated integrin β1 and NF-κB signaling pathways. In addition, *in vivo* results revealed that moderate mechanical stimulation could recover the water content of the NP and accelerate the reconstruction of the degenerative discs. Taken together, this study may foster progress towards understanding of the anti-inflammatory effects physiologically equivalent stimulations have on the cells and also provide the possibility of innovative design of physical therapies for DDDs. Further translational studies are needed to verify the clinical efficacy of mechanical stimulation by means of dynamic traction for DDD treatments.

## Supplementary Material

Supplementary figures.

## Figures and Tables

**Table 1:** Primers for qPCR

Gene	Forward (5'-3')	Reverse (5'-3')
*Cox2*	TTCCAGTATCAGAACCGCATTGCC	CCGTGTTCAAGGAGGATGGAGTTG
*Tnfa*	AAAGGACACCATGAGCACGGAAAG	CGCCACGAGCAGGAATGAGAAC
*Ilb*	TGTTTCCCTCCCTGCTGAC	CGACAATGCCTCGTGACC
*Il6*	ACTTCCAGCCAGTTGCCTTCTTG	TGGTCTGTTGTGGGTGGTATCCTC
*Gapdh*	GACATGCCGCCTGGAGAAAC	AGCCCAGGATGCCCTTTAGT

**Table 2:** Primary antibodies used in this study

Antibody	Vendor	Catalog number
Cav1	Cell Signaling Technology, MA, USA	3238
Cav1	Abcam, Cambridge, UK	ab17052
Integrin β1	Novus Biologicals, CO, USA	NBP2-16974
p65	Abcam, Cambridge, UK	ab32536
p-p65	ABclonal, MA, USA	AP0124
GAPDH	ABclonal, MA, USA	AC002
Collagen I	Abcam, Cambridge, UK	ab34710
Collagen II	Novus Biologicals, CO, USA	NB600-844
Aggrecan	GeneTex, CA, USA	GTX17497
COX-2	HuaBio, Zhejiang, China	RT1159
IgG	Cell Signaling Technology, MA, USA	3900

## Abbreviations

IVD: intervertebral disc
DDD: degenerative disc disease
YLDs: years lived with disability
AF: annulus fibrosus
AFC: annulus fibrosus cell
NP: nucleus pulposus
CEP: cartilage endplate
ECM: extracellular matrix
CTS: cyclic tensile strain
Cav1: Caveolin-1
Cav2: Caveolin-2
Cav3: Caveolin-3
NF-κB: Nuclear factor-kappa B
COX-2: Cyclooxygenase 2
IL-1β: Interleukin-1β
IL-6: Interleukin-6
IL-8: interleukin-8
DAPI: 4',6-diamidino-2-phenylindole
PDMS: polydimethylsiloxane
PVDF: polyvinylidene fluroride
PBS: phosphate buffered saline
FBS: fetal bovine serum
SDS-PAGE: sodium dodecyl sulfate polyacrylamide gel electrophoresis
BSA: bovine serum albumin
DMEM/F12: Dulbecco's modified eagle's medium, nutrient mixture F-12
H&E: hematoxylin & eosin
S.O.: Safranin O-Fast green
GO: gene ontology
KEGG: Kyoto encyclopedia of genes and genomes
EDTA: ethylene diamine tetraacetic acid
qPCR: quantitative polymerase chain reaction
SD: standard deviation
ANOVA: analysis of variance
Co-IP: co-immunoprecipitation
WCL: whole cell lysate

## References

[1] Hall JA, Konstantinou K, Lewis M, Oppong R, Ogollah R, Jowett S (2019). Systematic review of decision analytic modelling in economic evaluations of low back pain and sciatica. Appl Heath Econ Hea.

[2] Disease GBD, Injury I, Prevalence C (2016). Global, regional, and national incidence, prevalence, and years lived with disability for 310 diseases and injuries, 1990-2015: a systematic analysis for the Global Burden of Disease Study 2015. Lancet.

[3] Freburger JK, Holmes GM, Agans RP, Jackman AM, Darter JD, Wallace AS, Castel LD, Kalsbeek WD, Carey TS (2009). The rising prevalence of chronic low back pain. Arch Intern Med.

[4] Al-Riyami K, Voo S, Gnanasegaran G, Pressney I, Meir A, Casey A, Molloy S, Allibone J, Bomanji J (2019). The role of bone SPECT/CT in patients with persistent or recurrent lumbar pain following lumbar spine stabilization surgery. Eur J Nucl Med Mol Imaging.

[5] Gautschi OP, Stienen MN, Schaller K (2013). Spontaneous regression of lumbar and cervical disc herniations - a well established phenomenon. Praxis (Bern 1994).

[6] Choi YS (2009). Pathophysiology of degenerative disc disease. Asian Spine J.

[7] Finch P (2006). Technology Insight: imaging of low back pain. Nat Clin Pract Rheumatol.

[8] Yamazaki S, Banes AJ, Weinhold PS, Tsuzaki M, Kawakami M, Minchew JT (2002). Vibratory loading decreases extracellular matrix and matrix metalloproteinase gene expression in rabbit annulus cells. Spine J.

[9] Pratsinis H, Papadopoulou A, Neidlinger-Wilke C, Brayda-Bruno M, Wilke HJ, Kletsas D (2016). Cyclic tensile stress of human annulus fibrosus cells induces MAPK activation: involvement in proinflammatory gene expression. Osteoarthritis Cartilage.

[10] Khan AN, Jacobsen HE, Khan J, Filippi CG, Levine M, Lehman RA Jr, Riew KD, Lenke LG, Chahine NO (2017). Inflammatory biomarkers of low back pain and disc degeneration: a review. Ann N Y Acad Sci.

[11] Shamji MF, Setton LA, Jarvis W, So S, Chen J, Jing L, Bullock R, Isaacs RE, Brown C, Richardson WJ (2010). Proinflammatory cytokine expression profile in degenerated and herniated human intervertebral disc tissues. Arthritis Rheum.

[12] Santos SG, Lamghari M, Almeida CR, Oliveira MI, Neves N, Ribeiro AC, Barbosa JN, Barros R, Maciel J, Martins MC, Goncalves RM, Barbosa MA (2013). Adsorbed fibrinogen leads to improved bone regeneration and correlates with differences in the systemic immune response. Acta Biomater.

[13] Sun Z, Zhang M, Zhao XH, Liu ZH, Gao Y, Samartzis D, Wang HQ, Luo ZJ (2013). Immune cascades in human intervertebral disc: the pros and cons. Int J Clin Exp Pathol.

[14] Roberts S, Evans H, Trivedi J, Menage J (2006). Histology and pathology of the human intervertebral disc. J Bone Joint Surg Am.

[15] Gawri R, Rosenzweig DH, Krock E, Ouellet JA, Stone LS, Quinn TM, Haglund L (2014). High mechanical strain of primary intervertebral disc cells promotes secretion of inflammatory factors associated with disc degeneration and pain. Arthritis Res Ther.

[16] Rannou F, Richette P, Benallaoua M, Francois M, Genries V, Korwin-Zmijowska C, Revel M, Corvol M, Poiraudeau S (2003). Cyclic tensile stretch modulates proteoglycan production by intervertebral disc annulus fibrosus cells through production of nitrite oxide. J Cell Biochem.

[17] Urban JP, Roberts S (2003). Degeneration of the intervertebral disc. Arthritis Res Ther.

[18] Neidlinger-Wilke C, Galbusera F, Pratsinis H, Mavrogonatou E, Mietsch A, Kletsas D, Wilke HJ (2014). Mechanical loading of the intervertebral disc: from the macroscopic to the cellular level. Eur Spine J.

[19] Chen X, Yan J, He F, Zhong D, Yang H, Pei M, Luo ZP (2018). Mechanical stretch induces antioxidant responses and osteogenic differentiation in human mesenchymal stem cells through activation of the AMPK-SIRT1 signaling pathway. Free Radic Biol Med.

[20] Gawri R, Moir J, Ouellet J, Beckman L, Steffen T, Roughley P, Haglund L (2014). Physiological loading can restore the proteoglycan content in a model of early IVD degeneration. PLoS One.

[21] Schnake KJ, Putzier M, Haas NP, Kandziora F (2006). Mechanical concepts for disc regeneration. Eur Spine J.

[22] Pellecchia GL (1994). Lumbar traction: a review of the literature. J Orthop Sports Phys Ther.

[23] Kroeber M, Unglaub F, Guehring T, Nerlich A, Hadi T, Lotz J, Carstens C (2005). Effects of controlled dynamic disc distraction on degenerated intervertebral discs: an *in vivo* study on the rabbit lumbar spine model. Spine (Phila Pa 1976).

[24] Barczyk M, Carracedo S, Gullberg D (2010). Integrins. Cell Tissue Res.

[25] Zhang Y, Wang H (2012). Integrin signalling and function in immune cells. Immunology.

[26] Yan Z, Pan Y, Wang S, Cheng M, Kong H, Sun C, Hu K, Chen T, Dong Q, Chen J (2017). Static compression induces ECM remodeling and integrin alpha2beta1 expression and signaling in a rat tail caudal intervertebral disc degeneration model. Spine (Phila Pa 1976).

[27] Sasamoto A, Nagino M, Kobayashi S, Naruse K, Nimura Y, Sokabe M (2005). Mechanotransduction by integrin is essential for IL-6 secretion from endothelial cells in response to uniaxial continuous stretch. Am J Physiol Cell Physiol.

[28] Gabay C, Lamacchia C, Palmer G (2010). IL-1 pathways in inflammation and human diseases. Nat Rev Rheumatol.

[29] Wang Z, Hutton WC, Yoon ST (2014). Bone morphogenetic protein-7 antagonizes tumor necrosis factor-alpha-induced activation of nuclear factor kappaB and up-regulation of the ADAMTS, leading to decreased degradation of disc matrix macromolecules aggrecan and collagen II. Spine J.

[30] Deschner J, Hofman CR, Piesco NP, Agarwal S (2003). Signal transduction by mechanical strain in chondrocytes. Curr Opin Clin Nutr Metab Care.

[31] Xiao L, Xu HG, Wang H, Liu P, Liu C, Shen X, Zhang T, Xu YM (2016). Intermittent Cyclic Mechanical Tension Promotes Degeneration of Endplate Cartilage via the Nuclear Factor-kappaB Signaling Pathway: an *in vivo* Study. Orthop Surg.

[32] Tisherman R, Coelho P, Phillibert D, Wang D, Dong Q, Vo N, Kang J, Sowa G (2016). NF-kappaB Signaling Pathway in Controlling Intervertebral Disk Cell Response to Inflammatory and Mechanical Stressors. Phys Ther.

[33] Yeh YC, Parekh AB (2015). Distinct structural domains of caveolin-1 independently regulate Ca2+ release-activated Ca2+ channels and Ca2+ microdomain-dependent gene expression. Mol Cell Biol.

[34] Yeh YC, Tang MJ, Parekh AB (2014). Caveolin-1 alters the pattern of cytoplasmic Ca2+ oscillations and Ca2+-dependent gene expression by enhancing leukotriene receptor desensitization. J Biol Chem.

[35] Quest AF, Leyton L, Parraga M (2004). Caveolins, caveolae, and lipid rafts in cellular transport, signaling, and disease. Biochem Cell Biol.

[36] Sohn J, Brick RM, Tuan RS (2016). From embryonic development to human diseases: The functional role of caveolae/caveolin. Birth Defects Res C Embryo Today.

[37] Faggi F, Chiarelli N, Colombi M, Mitola S, Ronca R, Madaro L, Bouche M, Poliani PL, Vezzoli M, Longhena F, Monti E, Salani B, Maggi D, Keller C, Fanzani A (2015). Cavin-1 and Caveolin-1 are both required to support cell proliferation, migration and anchorage-independent cell growth in rhabdomyosarcoma. Lab Invest.

[38] Campbell L, Al-Jayyoussi G, Gutteridge R, Gumbleton N, Griffiths R, Gumbleton S, Smith MW, Griffiths DF, Gumbleton M (2013). Caveolin-1 in renal cell carcinoma promotes tumour cell invasion, and in co-operation with pERK predicts metastases in patients with clinically confined disease. J Transl Med.

[39] Goedicke-Fritz S, Kaistha A, Kacik M, Markert S, Hofmeister A, Busch C, Banfer S, Jacob R, Grgic I, Hoyer J (2015). Evidence for functional and dynamic microcompartmentation of Cav-1/TRPV4/K(Ca) in caveolae of endothelial cells. Eur J Cell Biol.

[40] Patel HH, Murray F, Insel PA (2008). Caveolae as organizers of pharmacologically relevant signal transduction molecules. Annu Rev Pharmacol Toxicol.

[41] Shihata WA, Michell DL, Andrews KL, Chin-Dusting JP (2016). Caveolae: a role in endothelial inflammation and mechanotransduction?. Front Physiol.

[42] Smolders LA, Meij BP, Onis D, Riemers FM, Bergknut N, Wubbolts R, Grinwis GC, Houweling M, Groot Koerkamp MJ, van Leenen D, Holstege FC, Hazewinkel HA, Creemers LB, Penning LC, Tryfonidou MA (2013). Gene expression profiling of early intervertebral disc degeneration reveals a down-regulation of canonical Wnt signaling and caveolin-1 expression: implications for development of regenerative strategies. Arthritis Res Ther.

[43] Yu L, Cai Y, Wang H, Pan L, Li J, Chen S, Liu Z, Han F, Li B (2020). Biomimetic bone regeneration using angle-ply collagen membrane-supported cell sheets subjected to mechanical conditioning. Acta Biomater.

[44] Yeh YC, Ling JY, Chen WC, Lin HH, Tang MJ (2017). Mechanotransduction of matrix stiffness in regulation of focal adhesion size and number: reciprocal regulation of caveolin-1 and beta1 integrin. Sci Rep.

[45] Chan SC, Ferguson SJ, Gantenbein-Ritter B (2011). The effects of dynamic loading on the intervertebral disc. Eur Spine J.

[46] Celtikci E, Yakar F, Celtikci P, Izci Y (2018). Relationship between individual payload weight and spondylolysis incidence in Turkish land forces. Neurosurg Focus.

[47] Shimozaki K, Nakase J, Yoshioka K, Takata Y, Asai K, Kitaoka K, Tsuchiya H (2018). Incidence rates and characteristics of abnormal lumbar findings and low back pain in child and adolescent weightlifter: A prospective three-year cohort study. PLoS One.

[48] Neidlinger-Wilke C, Wurtz K, Urban JP, Borm W, Arand M, Ignatius A, Wilke HJ, Claes LE (2006). Regulation of gene expression in intervertebral disc cells by low and high hydrostatic pressure. Eur Spine J.

[49] Butler DL, Goldstein SA, Guldberg RE, Guo XE, Kamm R, Laurencin CT, McIntire LV, Mow VC, Nerem RM, Sah RL, Soslowsky LJ, Spilker RL, Tranquillo RT (2009). The impact of biomechanics in tissue engineering and regenerative medicine. Tissue Eng Part B Rev.

[50] Cezar CA, Roche ET, Vandenburgh HH, Duda GN, Walsh CJ, Mooney DJ (2016). Biologic-free mechanically induced muscle regeneration. Proc Natl Acad Sci U S A.

[51] McNulty AL, Guilak F (2015). Mechanobiology of the meniscus. J Biomech.

[52] Robling AG, Niziolek PJ, Baldridge LA, Condon KW, Allen MR, Alam I, Mantila SM, Gluhak-Heinrich J, Bellido TM, Harris SE, Turner CH (2008). Mechanical stimulation of bone *in vivo* reduces osteocyte expression of Sost/sclerostin. J Biol Chem.

[53] Hasaneen NA, Zucker S, Cao J, Chiarelli C, Panettieri RA, Foda HD (2005). Cyclic mechanical strain-induced proliferation and migration of human airway smooth muscle cells: role of EMMPRIN and MMPs. FASEB J.

[54] Mousavi SJ, Doweidar MH (2015). Role of mechanical cues in cell differentiation and proliferation: a 3D numerical model. PLoS One.

[55] Janmey PA, McCulloch CA (2007). Cell mechanics: integrating cell responses to mechanical stimuli. Annu Rev Biomed Eng.

[56] Yoshigi M, Clark EB, Yost HJ (2003). Quantification of stretch-induced cytoskeletal remodeling in vascular endothelial cells by image processing. Cytometry A.

[57] Weinreich J, Agren MS, Bilali E, Kleinman HK, Coerper S, Konigsrainer A, Beckert S (2010). Effects of isoniazid and niacin on experimental wound-healing. Surgery.

[58] Midwood KS, Williams LV, Schwarzbauer JE (2004). Tissue repair and the dynamics of the extracellular matrix. Int J Biochem Cell Biol.

[59] Zhang Y, He Y, Bharadwaj S, Hammam N, Carnagey K, Myers R, Atala A, Van Dyke M (2009). Tissue-specific extracellular matrix coatings for the promotion of cell proliferation and maintenance of cell phenotype. Biomaterials.

[60] Luu YK, Capilla E, Rosen CJ, Gilsanz V, Pessin JE, Judex S, Rubin CT (2009). Mechanical stimulation of mesenchymal stem cell proliferation and differentiation promotes osteogenesis while preventing dietary-induced obesity. J Bone Miner Res.

[61] Thompson CL, Fu S, Knight MM, Thorpe SD (2020). Mechanical Stimulation: A Crucial Element of Organ-on-Chip Models. Front Bioeng Biotechnol.

[62] Iwashina T, Mochida J, Miyazaki T, Watanabe T, Iwabuchi S, Ando K, Hotta T, Sakai D (2006). Low-intensity pulsed ultrasound stimulates cell proliferation and proteoglycan production in rabbit intervertebral disc cells cultured in alginate. Biomaterials.

[63] Miyamoto K, An HS, Sah RL, Akeda K, Okuma M, Otten L, Thonar EJ, Masuda K (2005). Exposure to pulsed low intensity ultrasound stimulates extracellular matrix metabolism of bovine intervertebral disc cells cultured in alginate beads. Spine (Phila Pa 1976).

[64] Wenger KH, Woods JA, Holecek A, Eckstein EC, Robertson JT, Hasty KA (2005). Matrix remodeling expression in anulus cells subjected to increased compressive load. Spine (Phila Pa 1976).

[65] Su SC, Tanimoto K, Tanne Y, Kunimatsu R, Hirose N, Mitsuyoshi T, Okamoto Y, Tanne K (2014). Celecoxib exerts protective effects on extracellular matrix metabolism of mandibular condylar chondrocytes under excessive mechanical stress. Osteoarthritis Cartilage.

[66] Asakawa-Tanne Y, Su S, Kunimatsu R, Hirose N, Mitsuyoshi T, Okamoto Y, Tanaka E, Tanne K, Tanimoto K (2015). Effects of enzymatic degradation after loading in temporomandibular joint. J Dent Res.

[67] Hirose N, Okamoto Y, Yanoshita M, Asakawa Y, Sumi C, Takano M, Nishiyama S, Su SC, Mitsuyoshi T, Kunimatsu R, Tanne K, Tanimoto K (2020). Protective effects of cilengitide on inflammation in chondrocytes under excessive mechanical stress. Cell Biol Int.

[68] Fu S, Thompson CL, Ali A, Wang W, Chapple JP, Mitchison HM, Beales PL, Wann AKT, Knight MM (2019). Mechanical loading inhibits cartilage inflammatory signalling via an HDAC6 and IFT-dependent mechanism regulating primary cilia elongation. Osteoarthritis Cartilage.

[69] Griffin TM, Guilak F (2005). The role of mechanical loading in the onset and progression of osteoarthritis. Exerc Sport Sci Rev.

[70] Xu HT, Lee CW, Li MY, Wang YF, Yung PS, Lee OK (2020). The shift in macrophages polarisation after tendon injury: A systematic review. J Orthop Translat.

[71] Chow DHK, Yuen EMK, Xiao L, Leung MCP (2017). Mechanical effects of traction on lumbar intervertebral discs: A magnetic resonance imaging study. Musculoskelet Sci Pract.

[72] Sowa G, Agarwal S (2008). Cyclic tensile stress exerts a protective effect on intervertebral disc cells. Am J Phys Med Rehabil.

[73] Mui KL, Chen CS, Assoian RK (2016). The mechanical regulation of integrin-cadherin crosstalk organizes cells, signaling and forces. J Cell Sci.

[74] Tzima E, Irani-Tehrani M, Kiosses WB, Dejana E, Schultz DA, Engelhardt B, Cao G, DeLisser H, Schwartz MA (2005). A mechanosensory complex that mediates the endothelial cell response to fluid shear stress. Nature.

[75] Yamamoto H, Ehling M, Kato K, Kanai K, van Lessen M, Frye M, Zeuschner D, Nakayama M, Vestweber D, Adams RH (2015). Integrin beta1 controls VE-cadherin localization and blood vessel stability. Nat Commun.

[76] Toh YC, Xing J, Yu H (2015). Modulation of integrin and E-cadherin-mediated adhesions to spatially control heterogeneity in human pluripotent stem cell differentiation. Biomaterials.

[77] Parton RG (2018). Caveolae: structure, function, and relationship to disease. Annu Rev Cell Dev Biol.

[78] Chai Q, Wang XL, Zeldin DC, Lee HC (2013). Role of caveolae in shear stress-mediated endothelium-dependent dilation in coronary arteries. Cardiovasc Res.

[79] Gervasio OL, Phillips WD, Cole L, Allen DG (2011). Caveolae respond to cell stretch and contribute to stretch-induced signaling. J Cell Sci.

[80] Dubroca C, Loyer X, Retailleau K, Loirand G, Pacaud P, Feron O, Balligand JL, Levy BI, Heymes C, Henrion D (2007). RhoA activation and interaction with Caveolin-1 are critical for pressure-induced myogenic tone in rat mesenteric resistance arteries. Cardiovasc Res.

[81] Heathfield SK, Le Maitre CL, Hoyland JA (2008). Caveolin-1 expression and stress-induced premature senescence in human intervertebral disc degeneration. Arthritis Res Ther.

[82] Knapik DM, Perera P, Nam J, Blazek AD, Rath B, Leblebicioglu B, Das H, Wu LC, Hewett TE, Agarwal SK Jr, Robling AG, Flanigan DC, Lee BS, Agarwal S (2014). Mechanosignaling in bone health, trauma and inflammation. Antioxid Redox Signal.

[83] Che YJ, Guo JB, Liang T, Chen X, Zhang W, Yang HL, Luo ZP (2019). Controlled immobilization-traction based on intervertebral stability is conducive to the regeneration or repair of the degenerative disc: an *in vivo* study on the rat coccygeal model. Spine J.

[84] Guo JB, Che YJ, Hou JJ, Liang T, Zhang W, Lu Y, Yang HL, Luo ZP (2020). Stable mechanical environments created by a low-tension traction device is beneficial for the regeneration and repair of degenerated intervertebral discs. Spine J.

