# Supplementary material for: Moderate mechanical stimulation rescues degenerative annulus fibrosus by suppressing caveolin-1 mediated pro-inflammatory signaling pathway
Source: Int J Biol Sci. 2021 Apr 3;17(5):1395–412. doi: 10.7150/ijbs.57774 (PMC8040478; doi:10.7150/ijbs.57774)
Supplement: Supplementary file 1 — Supplementary figures. [file ijbsv17p1395s1.pdf]

Supplementary Materials

Figure S1

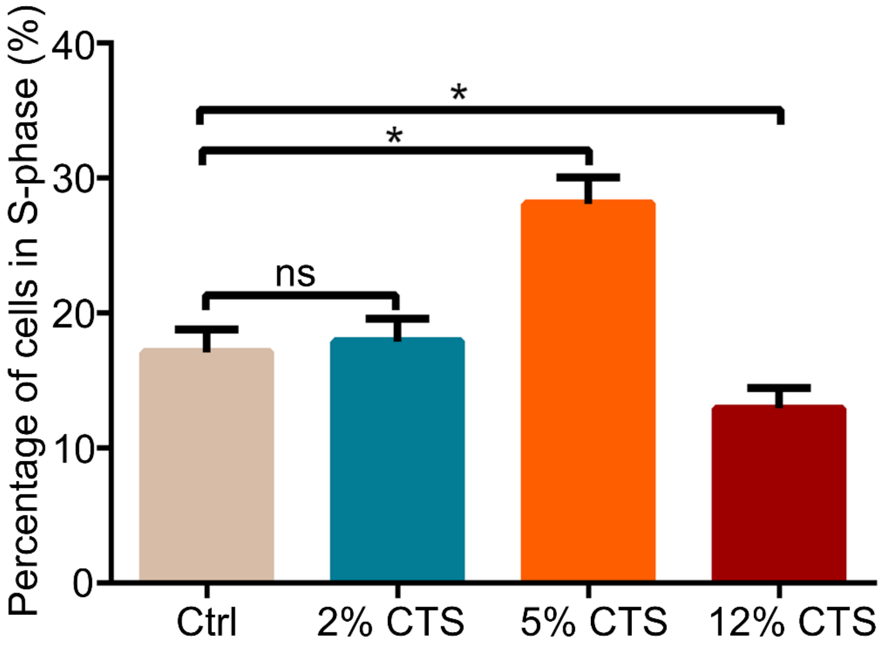

Figure S1. Percentage of S phase in cells under different mechanical conditions. (*$p<0.05$, $^{ns}p>0.05$ vs. Ctrl). The error bars indicate SD. N = 3.

Figure S2

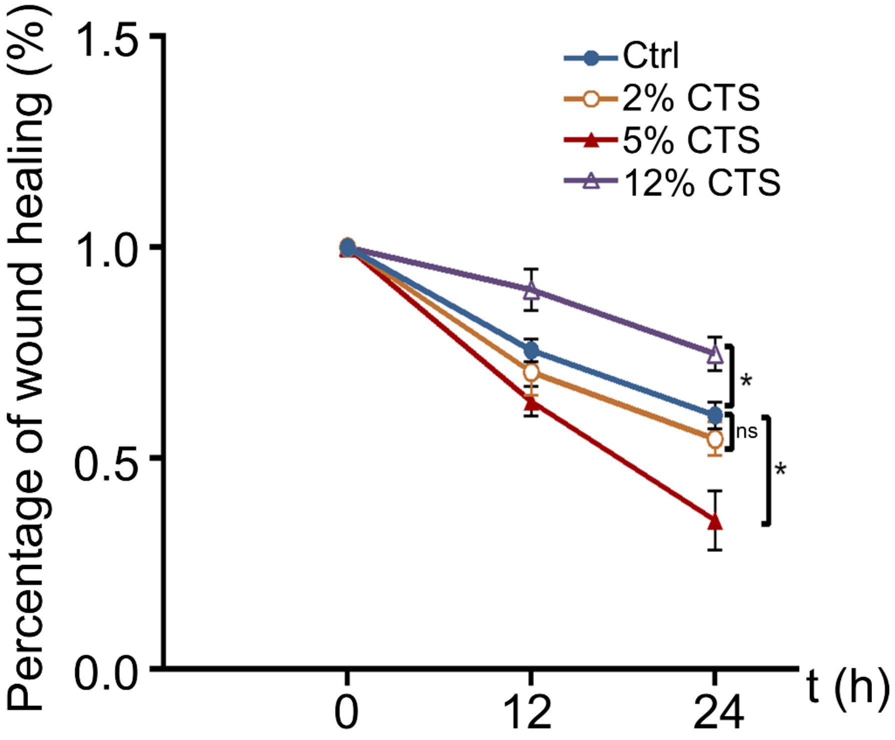

Figure S2. Wound closure expressed as the remaining area uncovered by the cells. The scratch area at time point 0 hours was set to 1. (*p<0.05, ^{ns}p>0.05 vs. Ctrl). The error bars indicate SD. N = 3.

Figure S3

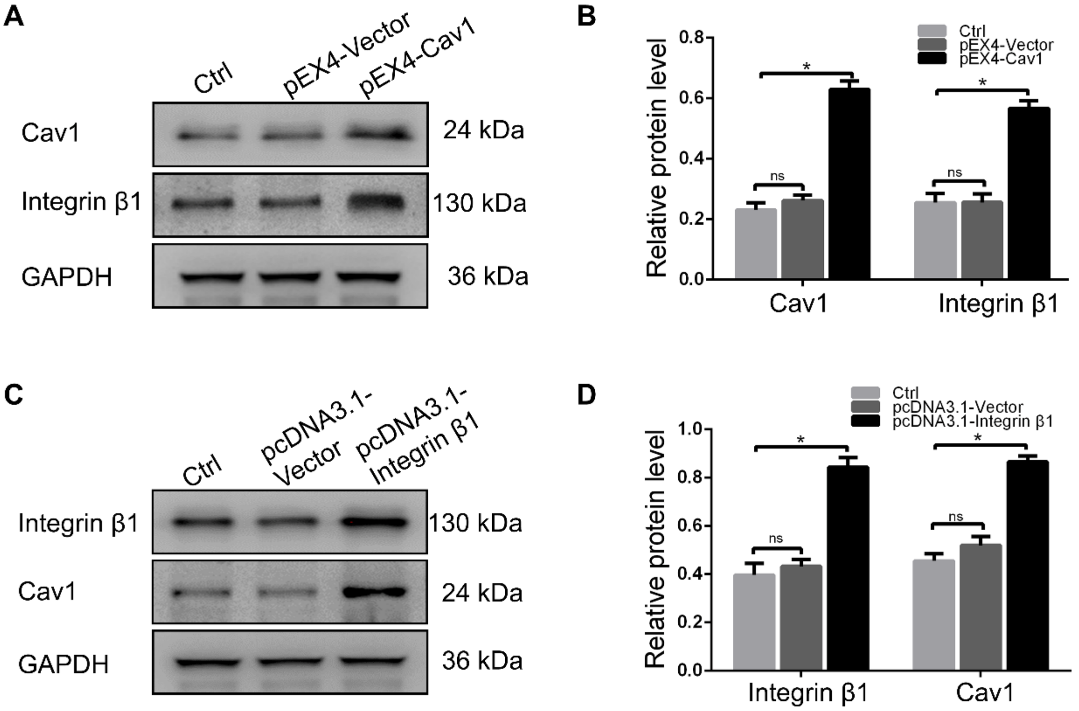

Figure S3. The interaction between Cav1 and integrin β1. **(A, B)** Western blot analysis of integrin β1 in AFCs after overexpression of Cav1. **(C, D)** Western blot analysis of Cav1 in AFCs after overexpression of integrin β1. (*p<0.05, ^{ns}p>0.05 vs. Ctrl). The error bars indicate SD. N = 3.

Figure S4

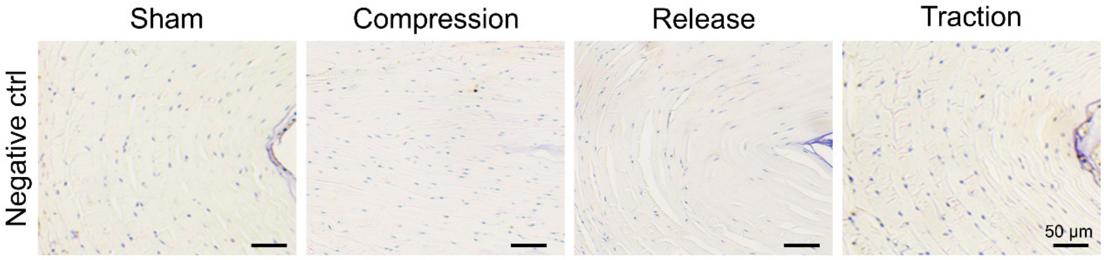

Figure S4. Negative control of immunohistochemistry for each group. N = 3. Scale bar = 50 μm.
